# Genetic variability of aquaporin expression in maize: From eQTLs to a MITE insertion regulating *PIP2;5* expression

**DOI:** 10.1093/plphys/kiae326

**Published:** 2024-06-05

**Authors:** Laurie C Maistriaux, Maxime J Laurent, Linda Jeanguenin, Santiago Alvarez Prado, Joseph Nader, Claude Welcker, Alain Charcosset, François Tardieu, Stéphane D Nicolas, François Chaumont

**Affiliations:** Louvain Institute of Biomolecular Science and Technology, UCLouvain, 1348 Louvain-la-Neuve, Belgium; Louvain Institute of Biomolecular Science and Technology, UCLouvain, 1348 Louvain-la-Neuve, Belgium; Louvain Institute of Biomolecular Science and Technology, UCLouvain, 1348 Louvain-la-Neuve, Belgium; INRAE, LEPSE, Université de Montpellier, 34060 Montpellier, France; Louvain Institute of Biomolecular Science and Technology, UCLouvain, 1348 Louvain-la-Neuve, Belgium; INRAE, LEPSE, Université de Montpellier, 34060 Montpellier, France; INRAE, CNRS, AgroParisTech, GQE-Le Moulon, Université Paris-Saclay, 91190 Gif-sur-Yvette, France; INRAE, LEPSE, Université de Montpellier, 34060 Montpellier, France; INRAE, CNRS, AgroParisTech, GQE-Le Moulon, Université Paris-Saclay, 91190 Gif-sur-Yvette, France; Louvain Institute of Biomolecular Science and Technology, UCLouvain, 1348 Louvain-la-Neuve, Belgium

## Abstract

Plant aquaporins are involved in numerous physiological processes, such as cellular homeostasis, tissue hydraulics, transpiration, and nutrient supply, and are key players of the response to environmental cues. While varying expression patterns of aquaporin genes have been described across organs, developmental stages, and stress conditions, the underlying regulation mechanisms remain elusive. Hence, this work aimed to shed light on the expression variability of 4 plasma membrane intrinsic protein (*PIP*) genes in maize (*Zea mays*) leaves, and its genetic causes, through expression quantitative trait locus (eQTL) mapping across a 252-hybrid diversity panel. Significant genetic variability in *PIP* transcript abundance was observed to different extents depending on the isoforms. The genome-wide association study mapped numerous eQTLs, both local and distant, thus emphasizing the existing natural diversity of *PIP* gene expression across the studied panel and the potential to reveal regulatory actors and mechanisms. One eQTL associated with *PIP2;5* expression variation was characterized. Genomic sequence comparison and in vivo reporter assay attributed, at least partly, the local eQTL to a transposon-containing polymorphism in the *PIP2;5* promoter. This work paves the way to the molecular understanding of *PIP* gene regulation and its possible integration into larger networks regulating physiological and stress adaptation processes.

## Teaser

Quantitative study of the variability in aquaporin gene expression in maize leaves identifies several expression quantitative trait loci, one of which includes a transposon-containing polymorphism.

## Introduction

The vital need for water homeostasis places water channels or aquaporins as essential life molecular components taking part in numerous processes at the cell to organism scales. Their particular diversity in plants emphasizes their multiple important roles in the sessile autotrophic lifestyle. Aquaporins are involved in the control of the root and leaf hydraulic conductivity, transpiration, photosynthesis, and osmoregulation. Over the years, a tight and complex regulation of aquaporins belonging to the plasma membrane intrinsic protein (PIP) subfamily acting at multiple levels (abundance, trafficking, gating) was revealed (Chaumont and Tyerman 2014; Maurel et al. 2015).

In maize (*Zea mays*), with a few rare exceptions, expression of most of the identified 13 *PIP* genes is detected both in leaves and roots, although to different extents, and is associated with protein abundance for isoforms for which specific antibodies were available (Hachez et al. 2006, 2008). In both organs, expression varies along the longitudinal development and displays diurnal or circadian variation (Lopez et al. 2003; Hachez et al. 2006, 2008; Caldeira et al. 2014). In leaves, *PIP* expression is generally low at the base and gradually increases in the elongation zone (EZ) to reach a peak where the leaf emerges from the sheath and the leaf surface becomes exposed to the atmosphere, increasing the evaporative demand from the xylem (Hachez et al. 2008). The expression of certain isoforms, such as *PIP1;1* and *PIP1;3*, plateaued or slightly decreases in the mature zone (MZ) of the leaf. In contrast, *PIP1;2* and *PIP2;1*, *PIP2;3*, *PIP2;4*, and *PIP2;5* exhibit a bell-shaped expression pattern, decreasing in the MZ. Transcript levels of *PIP1;4*, *PIP1;6*, *PIP2;2*, and *PIP2;6* remain relatively constant along the leaf longitudinal axis (Hachez et al. 2008). In addition, *PIP* expression in the EZ is quite stable with the time of the day, whereas in the MZ, almost all the *PIP* genes show a similar diurnal expression pattern with high expression in the morning, a drop in levels during the day, and a low level during the night. Together with immunocytochemistry data, these results suggest the importance of a tight regulation of *PIP* gene expression in specific leaf cells to account for water radial movement, in particular in vascular bundles and the mesophyll cells during evapotranspiration (Hachez et al. 2008; Heinen et al. 2009). Furthermore, a role of PIP aquaporins in maize stomatal dynamics was also demonstrated (Heinen et al. 2014; Ding et al. 2022). For instance, *PIP2;5* is expressed in guard cells of the stomatal complexes, and its over- and down-expression results in faster or slower stomatal closure upon abscisic acid treatment, respectively, compared with wild-type plants (Ding et al. 2022).

While the spatial and dynamic expression patterns of aquaporin genes across organs, developmental stages, and environmental conditions have been extensively described, the existing natural diversity in aquaporin expression among different cultivars or lines within a given species has hardly been assessed and exploited so far, except for rare studies in which the comparison between only a few genetically divergent accessions was driven by peculiar phenotypes (Vandeleur et al. 2009; Sutka et al. 2011; Devi et al. 2016; Khan et al. 2019; Pou et al. 2022; Rishmawi et al. 2023; Vaziriyeganeh et al. 2023). In addition, the molecular determinants and mechanisms responsible for aquaporin gene expression regulation are scarce.

Here, we shed light on the natural genetic variability of *PIP* gene expression in maize leaves among lines selected over decades, via a panel representing the diversity of cultivated maize (a 252-hybrid panel, gathered in the framework of the European Drought-Tolerant Yielding Plants [DROPS] project; Millet et al. 2016). We also explored to what extent the variability is the result of a robust genetic architecture by carrying out a genome-wide association study (GWAS) of the expression of 4 *PIP* genes assessed by reverse transcription quantitative PCR (RT-qPCR). Samples were collected in both EZ and MZ of the leaf, because of their different physiological states and major processes at stake (water uptake–mediated cell growth vs. photosynthesis and transpiration) and the putative differential implications of *PIP* genes in these processes. The 4 *PIP* genes were chosen based on particular interests for their known expression patterns and functional data: *PIP1;1* because of its ubiquity and a priori water channel inactivity (Fetter et al. 2004); *PIP1;3* and *PIP2;2* because of their relative high expression in leaves, including in the stomatal complexes (Hachez et al. 2008; Heinen et al. 2014); and *PIP2;5* because its basal expression in the stomatal complexes affects their closure dynamics (Ding et al. 2022). The resulting mapped eQTLs were mined for molecular determinants regulating *PIP* gene expression, based on eQTL physiognomy, available genomic resources and annotations, and relevant literature. Ultimately, molecular validation of a putative *cis*-acting local eQTL of *PIP2;5*, consisting in a miniature inverted repeat transposable element (MITE)−containing insertion in its promoter, was conducted by transactivation assay in maize cells. This work constitutes a first step toward elucidating the regulatory elements affecting *PIP* gene expression in maize.

## Results

### *PIP* gene expression varies across maize genetic groups

*PIP1;1*, *PIP1;3*, *PIP2;2*, and *PIP2;5* mRNA transcript levels (i.e. referred to as “expression”) were analyzed by RT-qPCR (primers in Supplementary Table S1), across a panel of 252 hybrids obtained by crossing 252 dent inbred lines with a common unrelated flint parent (UH007), a usual practice in hybrid crop genetics (Rincent et al. 2014a; Negro et al. 2019; Hu et al. 2022). As all hybrids share a common parent, the variation of mRNA abundance between hybrids depends on the variation of mRNA between lines, if we consider that there is no variation of specific interaction between lines and tester. We tested therefore at each locus if there was significant difference of expression between hybrids derived from lines, which shared a common allele with tester (homozygote), and hybrids from lines, which did not share alleles with tester (heterozygote). If the allele-specific expression (ASE) is defined as the imbalance of expression between 2 alleles at 1 locus (Cleary and Seoighe 2021), we tested if there was or not an ASE at each locus along the genome. In absence of ASE, we expected no difference between homozygote and heterozygote.

Relative expression data showed significant weak to moderate correlations between isoforms and between zones for most isoforms (Supplementary Fig. S1). The relative range of expression of the different isoforms (*PIP1;1* > *PIP1;3* and *PIP2;2* > *PIP2;5*) coincided with published results in leaves (Supplementary Fig. S2; Hachez et al. 2008). All traits (combinations of *PIP* isoform × leaf zone, hereafter referred to as *PIPX;X_*MZ/EZ) showed moderate to high heritability values, supporting the relevance of the genetic factor in the observed variations (Table 1). The lower value for *PIP2;5*_EZ may be explained by higher experimental noise due to low expression close to the detection threshold.

**Table 1.** “Heritability” of the *PIP* gene expression in both leaf zones

Leaf zone	*PIP1;1*	*PIP1;3*	*PIP2;2*	*PIP2;5*
EZ	0.567	0.821	0.936	0.419
MZ	0.848	0.798	0.887	0.728

It was calculated from a linear mixed model stating Expression ∼ Genotype (random) and as *V*_g_/(*V*_g_ + *V*_e_/*n*) where *V*_g_ is the variance attributed to the genotype, *V*_e_ is the residual variance, and *n* is the number of replicates.

**Figure 1.**
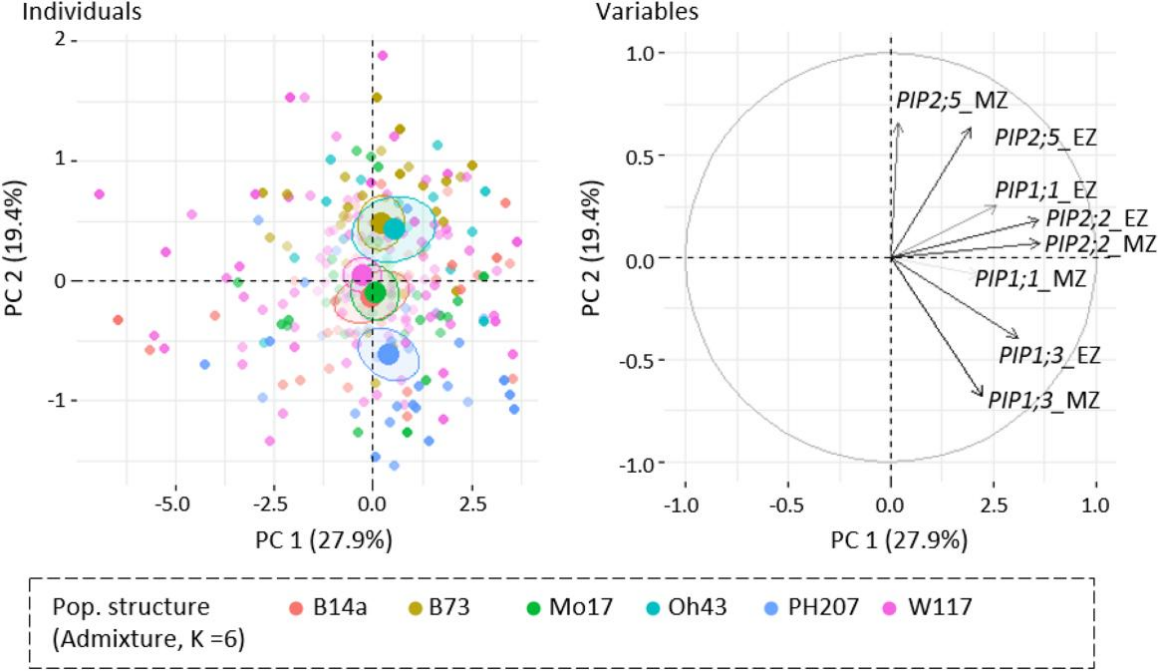
PCA of *PIP* expression data in the leaf MZ and EZ. On the left, the graph of individuals shows the different genotypes, colored according to their belonging to the defined genetic groups (admixture, *K* = 6), named according to their respective representative lines (B73, stiff stalk; B14a, stiff stalk early; Mo17, Lancaster; Oh43, Lancaster early; PH207, iodent; W117 admixture [W117, NC358, and C103]). Transparency of the points correlates with their quality of representation in the shown space (cos^2^ parameter; i.e. plain points are better represented on this particular space than the more transparent ones). On the right, the graph of variables shows the relationships of the explanatory variables, i.e. the 4 *PIP* isoform expression data, with the first and second dimensions. Similarly, the arrow transparency reflects their representation quality. PCA was performed with the PCA function in R and plotted with the factoextra package functions.

**Figure 2.**
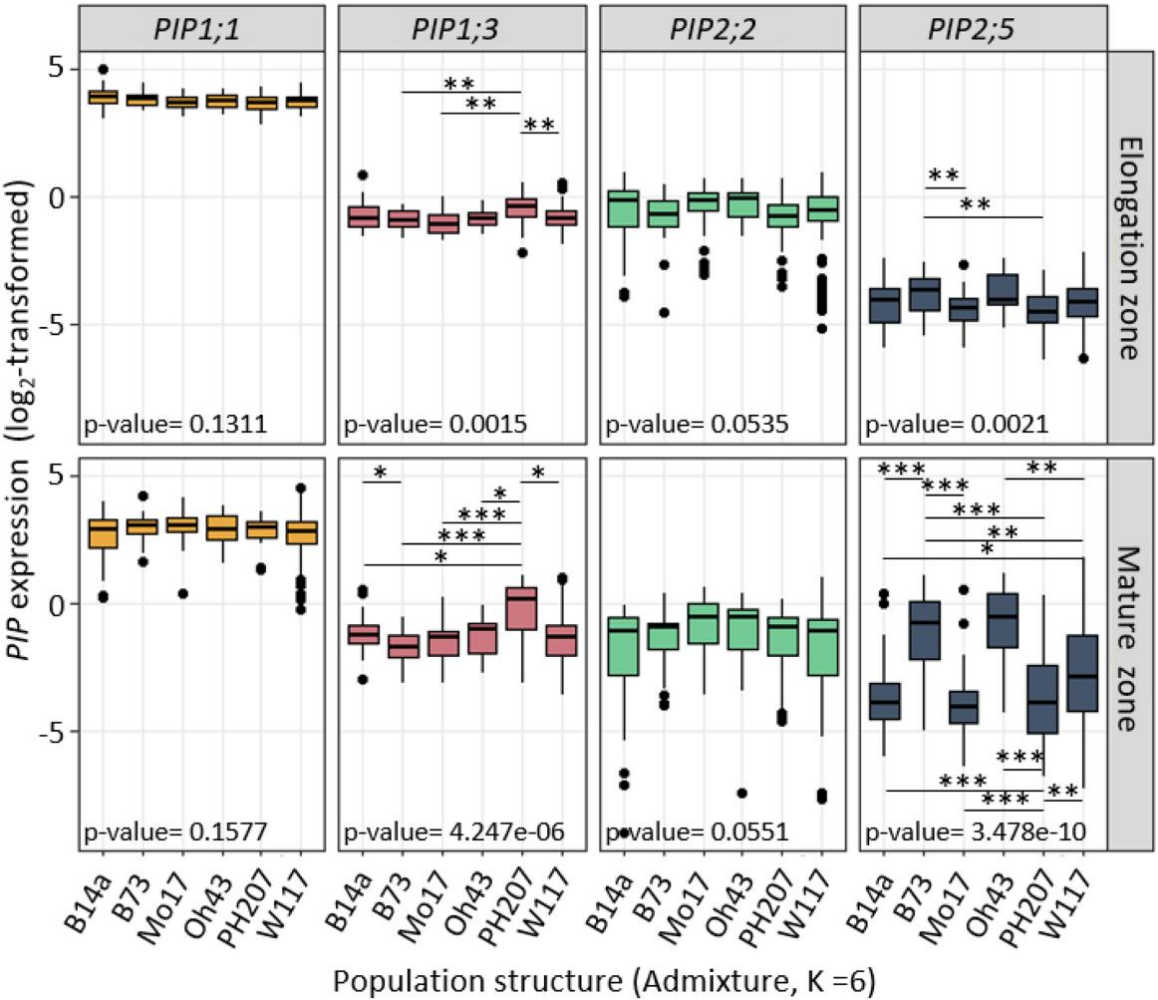
*PIP* gene expression data according to population structure groups. Relative expression data (log_2_ transformed) are shown for the 4 *PIP* isoforms and the 2 leaf zones, the EZ (above) and the MZ (below). Data for all lines are classified according to their belonging to the defined population structure groups (admixture, *K* = 6), named according to their respective representative lines. The *P*-values come from the nonparametric one-way Kruskal–Wallis test (kruskal.test R function), while * indicates the significant pairwise comparisons (pairwise.wilcox.test R function, Holm adjustment for multitesting *0.1, **0.05, and ***0.01). Population groups are defined as B73, stiff stalk; B14a, stiff stalk early; Mo17, Lancaster; Oh43, Lancaster early; PH207, iodent; and W117 admixture (W117, NC358, and C103). The center line in the boxplot indicates the relative expression median, points are outliers, and the box upper and lower limits are the third and first quartiles, respectively. The whiskers represent the 1.5× interquartile range.

Principal component (PC) analysis of *PIP* expression data (Fig. 1) revealed a partial isoform-specific correlation between the different leaf zones. However, the expressions between the different *PIP* genes were weakly correlated. Indeed, *PIP2;5* expression was strongly correlated with the second PC (capturing 19.4% of the variance), while other isoform expressions were generally more strongly associated with the first PC (27.9%), which could reflect the global expression of *PIP* isoforms. The second PC clearly discriminated PH207, B14a, and Mo17 genetic groups in the lower part from B73 and Oh43 groups in the upper part suggesting that *PIP* expression could depend on genetic groups. Furthermore, we revealed some significant differences in *PIP* expression according to genetic groups (Fig. 2). Among various group- and isoform-specific differences, the B73 and Oh43 groups showed remarkably higher *PIP2;5* expression in the MZ, with this trend being conserved with a smaller amplitude in the EZ (Fig. 2). In contrast, *PIP1;1* showed less global variation, especially in the EZ. The marked variability in *PIP2;5* expression confirmed what was highlighted in the PCA, in both the MZ and EZ.

### eQTL mapping by GWAS reveals local and distant eQTLs controlling *PIP* expression

**Figure 3.**
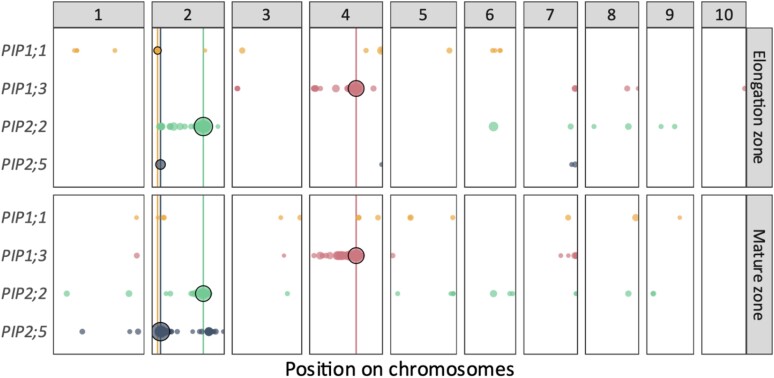
eQTLs detected for the *PIP* expression in the leaf EZ and MZ. Dot diameter is proportional to the −log_10_(*P*-value). This representation corresponds to a Manhattan plot viewed from the top. Chromosomes are indicated by numbers 1 to 10. Vertical lines indicate the position of the *PIP* genes of interest. The local eQTLs are circled in black. Physical positions of the markers are based on the B73 reference genome version 4. Significance threshold is set at 5.

**Figure 4.**
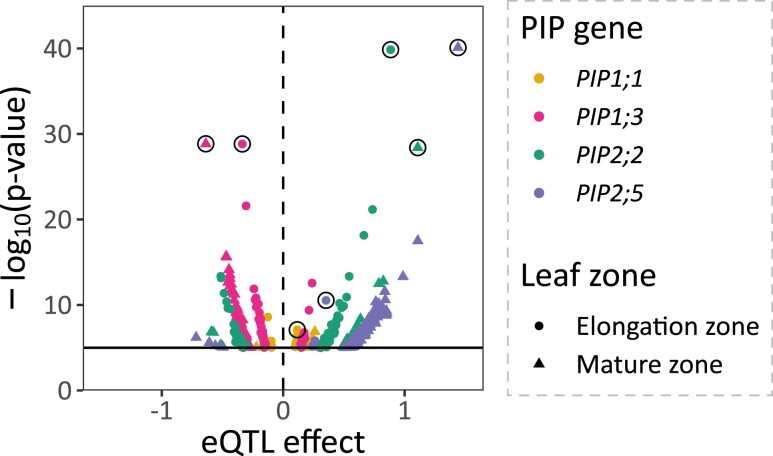
eQTL effect. Significance and effect of detected eQTLs, based on their representative lead SNP. Significance is represented as −log_10_(*P*-value), with a threshold set at 5. The eQTL effect was assessed using the B73 allele as reference. Local eQTLs are circled in black.

A GWAS with 737,202 single-nucleotide polymorphisms (SNPs) on expression of 4 *PIP* genes in EZ and MZ resulted in 3,490 significant associated SNPs (Fig. 3 and Table 2;Supplementary Table S2). We grouped these significant SNPs into 443 eQTLs based on the overlap of their estimated linkage disequilibrium (LD) windows (the so-called LD-win method; Negro et al. 2019). Local eQTLs were defined as containing the *PIP* gene of interest within their borders, while other eQTLs were termed distant eQTLs. Local and distant terms were preferred to *cis* and *trans* terms, which infer a biological mode of action. By using local and distant terms, we avoid making assumptions about their role (Blein-Nicolas et al. 2020). Local eQTLs were found for all traits but one, *PIP1;1*_MZ (circled in black in Figs. 3 and 4). In all but 1 case (*PIP1;1*_EZ), they appeared to be the most significant eQTLs and showed the largest effect (Fig. 4 andTable 2). Interestingly, local eQTLs associated with a given isoform in both leaf zones were not necessarily similar (*PIP1;1* and *PIP1;3*), suggesting that multiple regulatory elements with different impacts on *PIP* gene expression and spatial relevance are involved. In other cases, both EZ and MZ local eQTLs coincided either because they were described by the same lead SNP (*PIP2;2*) or because their respective lead SNPs showed the same direction effect, close proximity, and highly similar allele segregation across the panel (*PIP2;5*; Table 2), thus supporting the relevance of major regulation mechanisms acting regardless of the leaf zone. Besides local eQTLs, 436 distant eQTLs were also detected, distributed on all chromosomes (Fig. 3). Unlike local eQTLs, which were sometimes shared between the 2 leaf zones related to a unique *PIP* gene, 295 distant eQTLs were only found in a single leaf zone, suggesting a mostly isoform- and zone-specific regulation of *PIP* genes.

**Table 2.** eQTLs for the *PIP* genes with the initial GWAS model

Gene	Zone	SNP no.	QTL no.	Local?	Local eQTL								
					Top?	SNP no.	Range^a^	Lead SNP	LogPval^b^	*r* ^2^_LR_^c^	Effect	Position^a^	MAF^d^
*PIP1;1*	EZ	50	18	Yes	No	11	chr2:19315776 to 19353266	AX-90735145	7.1	0.1	0.1	chr2:19340946	0.34
	MZ	38	15	No	…		…	…	…	…	…	…	…
*PIP1;3*	EZ	754	65	Yes	Yes	78	chr4:157020802 to 157249685	AX-90895950	28.8	0.4	−0.3	chr4:157203881	0.48
	MZ	890	97	Yes	Yes	70	chr4:157020309 to 157249685	S4_153627019	28.8	0.4	−0.6	chr4:157200014	0.33
*PIP2;2*	EZ	505	75	Yes	Yes	18	chr2:173109023 to 173430699	PZE-102122092	39.9	0.5	0.9	chr2:173382757	0.11
	MZ	167	48	Yes	Yes	9	chr2:173109023 to 173430699	PZE-102122092	28.4	0.4	1.1	chr2:173382757	0.11
*PIP2;5*	EZ	36	4	Yes	Yes	32	chr2:29138193 to 29445124	S2_27871636	10.5	0.1	0.4	chr2:29279736	0.33
	MZ	936	112	Yes	Yes	128	chr2:29137698 to 29507155	AX-91209412^e^	40.1	0.5	1.4	chr2:29280131	0.32

For each trait, the number of significant SNPs and defined eQTLs is indicated. When a local eQTL was detected, its range, lead SNP, and associated features are presented. It is also indicated whether or not it is the most significant peak detected for this trait (“Top?”). Ellipsis indicates the absence of information.

^a^Physical positions refer to the B73 reference genome version 4.

^b^LogPval stands for −log10(*P*-value).

^c^The *r*^2^_LR_ estimates the percentage of variance explained by the lead SNP of the eQTL calculated as in Sun et al. (2010).

^d^MAF indicates the minor allele frequency of the given marker among the panel.

^e^Note that 3 redundant SNPs were detected as the most significant for the local *PIP2;5* eQTL (AX-91209412, AX-90737936, and AX-91514455). The first one, in terms of physical position (and relative position to *PIP2;5*), was chosen as the lead SNP.

### Covariate GWAS highlights new local eQTLs

For *PIP1;3* and *PIP2;5*, we observed many distant eQTLs clustered around their local eQTLs creating a large tail around them (Fig. 3). Since the effect of these local eQTLs was very high in the 2 leaf zones (e.g. *r*^2^_LR_ = 0.50 for *PIP2;5*; Table 2), large tails of significant associations around them certainly originated from the existence of moderate LD with the local ones and thus the same causal polymorphisms. Besides, significant associations were sometimes observed with distant eQTLs located far away on the same chromosome. It is noteworthy that such a phenomenon of moderate to high LD between very distant loci was already observed, even despite the correction for kinship, and may be explained by chromosomal rearrangement or strong epistatic interactions (Negro et al. 2019). Ultimately, the presence of eQTL tails around local eQTLs could hide the presence of eQTLs capturing the effect of other causal polymorphisms.

**Figure 5.**
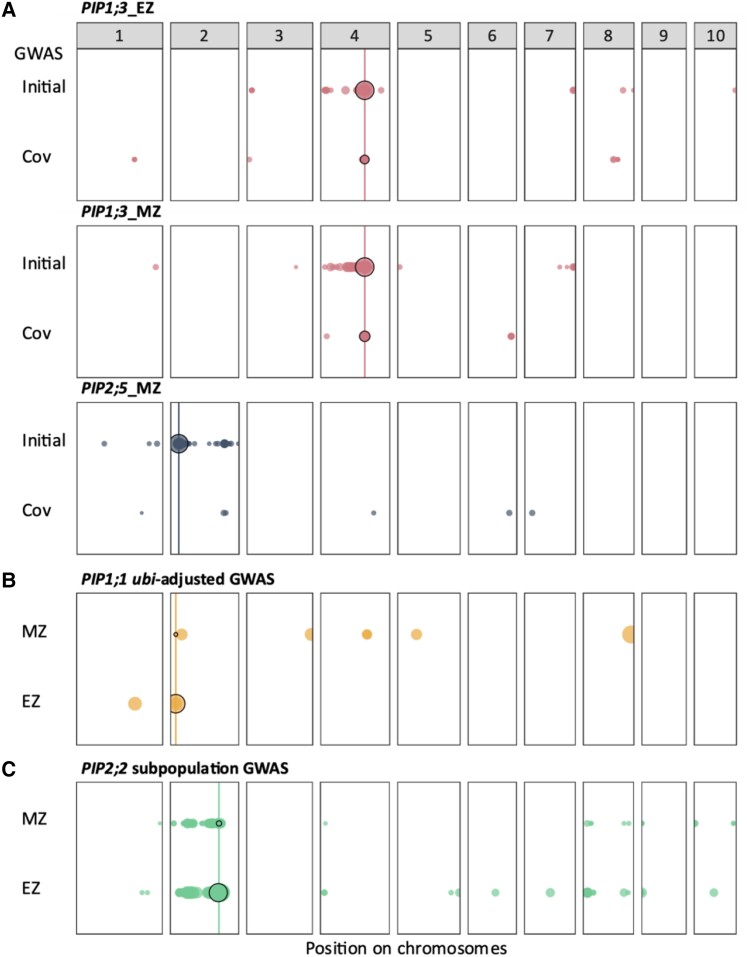
Covariate GWAS. **A)** When large local eQTL peaks were detected (arbitrary cutoff at −log_10_(*P*-value) > 12), covariate GWAS was performed by integrating the local lead SNP as additional covariate in the model. Both initial and covariate (“Cov”) GWAS are shown for the concerned data sets. **B)** Ubi-adjusted GWAS for *PIP1;1* traits. **C)** GWAS on subpopulation for *PIP2;2* (220 lines), due to the detection of a low conservation at the location of the expected forward primer RT-qPCR hybridization. Dot diameter is proportional to the −log10(*P*-value). Vertical lines indicate the position of the *PIP* genes of interest, thus defining the local eQTLs circled in black. Physical positions of the markers are based on the B73 reference genome version 4. Significance threshold is set at 5.

To solve this problem, we used a multilocus approach where we integrated the local eQTL lead SNP as a covariate in the GWAS to exclude all highly linked and redundant loci (referred to as “Cov”; Fig. 5A). This was implemented for traits with arbitrary large local eQTLs [with a −log_10_(*P*-value) > 12], i.e. *PIP1;3_EZ/MZ* and *PIP2;5*_MZ. As expected, this approach resulted in a large reduction in the number of associated SNP and QTL number (Table 3) and, in most cases, cleared the area around the local eQTL. All lost eQTLs may thus be considered with high caution as they were somehow linked to the local ones. On the other hand, this allowed the emergence of other eQTLs that could safely be considered as independent from the local ones. They showed globally lower −log_10_(*P*-value) than the initial ones. Interestingly, different local eQTLs were detected in some cases (*PIP1;3_EZ/MZ*), suggesting the presence of multiple causal polymorphisms in the close vicinity of the *PIP* genes (Fig. 5A).

**Table 3.** Final set of eQTLs detected for 4 AQP genes with different GWAS models with a focus on local eQTLs

Gene	Zone	GWAS	SNP no.	QTL no.	Local?	Top?	Local eQTL							
							SNP no.	Range^a^	Lead SNP	LogPval^b^	*r* ^2^_LR_^c^	Effect	Position^a^	MAF^d^
*PIP1;1*	*EZ*	*ubi*-adj	13	3	Yes	Yes	5	chr2:19315776 to 19353213	AX-90735145	6.66	0.12	0.10	chr2:19340946	0.34
	*MZ*	*ubi*-adj	17	7	Yes	No	1	chr2:19329871 to 19354321	S2_18132982	5.02	0.10	0.23	chr2:19342082	0.39
*PIP1;3*	*EZ*	Initial	754	65	Yes	Yes	78	chr4:157020802 to 157249685	AX-90895950	28.83	0.39	−0.34	chr4:157203881	0.48
		Cov	31	14	Yes	Yes	5	chr4:157173459 to 157250577	AX-91439361	7.71	0.2	−0.17	chr4:157117582	0.38
	*MZ*	Initial	889	97	Yes	Yes	70	chr4:157020309 to 157249685	S4_153627019	28.84	0.39	−0.64	chr4:157200014	0.34
		Cov	148	33	Yes	No	29	chr4:157173459 to 157250577	S4_153627596	9.77	0.2	−0.35	chr4:157200591	0.13
*PIP2;2*	*EZ*	Subpop	1673	161	Yes	…	132	chr2:167619354 to 173427207	AX-90774142	28.46	0.4	0.52	chr2:170958393	0.06
	*MZ*	Subpop	475	82	Yes	No	1	chr2:173330947 to 173427207	S2_168551532	5.23	0.08	0.37	chr2:173379237	0.08
*PIP2;5*	*EZ*	Initial	36	4	Yes	Yes	32	chr2:29138193 to 29445124	S2_27871636	10.54	0.2	0.35	chr2:29279736	0.33
	*MZ*	Initial	935	112	Yes	Yes	128	chr2:29137698 to 29507155	AX-91209412	40.11	0.50	1.44	chr2:29280131	0.32
		Cov	22	6	No	…	…	…	…	…	…	…	…	…

For each trait, the number of significant SNPs and defined eQTLs is indicated including the GWAS model used. When detected, characteristics of the local eQTL are presented. It is indicated whether or not it is the most significant peak detected for this trait (“Top?”). Ellipsis indicates the absence of information.

^a^Physical positions refer to the B73 reference genome version 4.

^b^LogPval stands for −log_10_(*P*-value).

^c^The *r*^2^_LR_ estimates the percentage of variance explained by the lead SNP of the eQTL calculated as in Sun et al. (2010).

^d^MAF indicates the minor allele frequency of the given marker among the panel.

### Limitations of eQTL detection due to qPCR technical aspects

Due to the intrinsic influence of RT-qPCR endogenous control genes in the expression measurements, the presence of any important eQTL in the vicinity of these genes was controlled for all the traits. Attention was drawn by an eQTL associated with *PIP1;1* expression in the EZ, colocalizing with the *UBIQUITIN 2* gene (*Ubi2*) targeted by the *Ubi* primer pair. This so-called *ubi*-eQTL was only detected for *PIP1;1* and was the most significant [−log_10_(*P*-value) = 8.59, lead SNP = AX-91641079] for *PIP1;1*_EZ (Supplementary Fig. S3, A to C). A clear bias was observed for *PIP1;1*_EZ expression data (and, to a lower extent, for *PIP1;1*_MZ) in light of this *ubi*-eQTL (Supplementary Fig. S3D). We then calculated *PIP1;1* expression using only *ACTIN 1* (*Act1*) and *ELONGATION FACTOR 1α* (*Ef1α*) as reference genes, and this variation disappeared (Supplementary Fig. S3E), and, after performing a GWAS, the *ubi*-eQTL disappeared, suggesting that it was an artifact (Supplementary Fig. S3F). However, in order to keep the expression data consistent for the other *PIP* genes, we took into account this experimental bias for both *PIP1;1* data sets by setting the lead SNP of this *ubi*-eQTL as covariate into the GWAS model leading to a new collection of eQTL (the so-called *ubi*-adjusted eQTLs; Fig. 5B). Interestingly, this new GWAS model allowed the recovering of a weak local eQTLs for *PIP1;1*_MZ that was previously the only data set not harboring one. The initial *PIP1;1*_EZ local eQTL remained, although slightly less significant [−log10(*P*-value) = 6.66 vs. 7.10]. It is noteworthy that no eQTL was detected for any other data sets around *ubiquitin* 2 and no noticeable eQTL was detected around the 2 other endogenous control genes (*Act1* and *Ef1α*; Supplementary Fig. S3).

Moreover, we investigated the presence of polymorphisms among the sequences targeted by the qPCR primers and evaluated the impact of such pinned polymorphisms on the eQTL detection (Supplementary Appendix S1). Three genotyped SNPs were identified in the primer pair targeting *PIP1;1*, but none of them seems to have an impact on the local eQTL detected (Supplementary Appendix S1). Regarding *PIP2;2*, genome and targeted sequence alignment revealed a very different sequence at the expected target site of the forward primer, and the local eQTL detected (identical lead SNP for MZ and EZ data sets) was strongly associated with the repartition of the alleles at this forward primer location. As the unfavorable allele concerned only 29 lines, we discarded them from the *PIP2;2* analysis, and only the 220 remaining ones, harboring the favorable allele, were used for the GWAS (3 line genotypes as heterozygous at this location were also discarded; Fig. 5C). For *PIP1;3*, 1 genotyped SNP within the forward primer was associated with the local eQTL obtained in the EZ, but not with the local eQTL detected in the MZ. However, multiple other causal polymorphisms seem to exist to explain the local eQTL in the EZ (Supplementary Appendix S1). Finally, no polymorphism was identified among the regions targeted by the RT-qPCR primers of the *PIP2;5* gene, either in the marker collection or when comparing genomes (Supplementary Appendix S1).

### Final eQTL set identifies a *PIP2;5* local eQTL

We ended up with a final set of eQTLs (Table 3; Supplementary Tables S3 and S4). In the cases where covariate GWAS was implemented, eQTLs coming from the initial GWAS but lost during the covariate GWAS were still conserved because a part of them may still reveal biologically relevant linkage between distant loci (recent relocation, transposition, co-evolution, epistasis). They should, however, be considered with the greatest caution. As for the initial GWAS results, we did not notice any striking colocalization between distant eQTLs detected for different *PIP* isoforms that could have pointed out at potential shared regulators.

**Figure 6.**
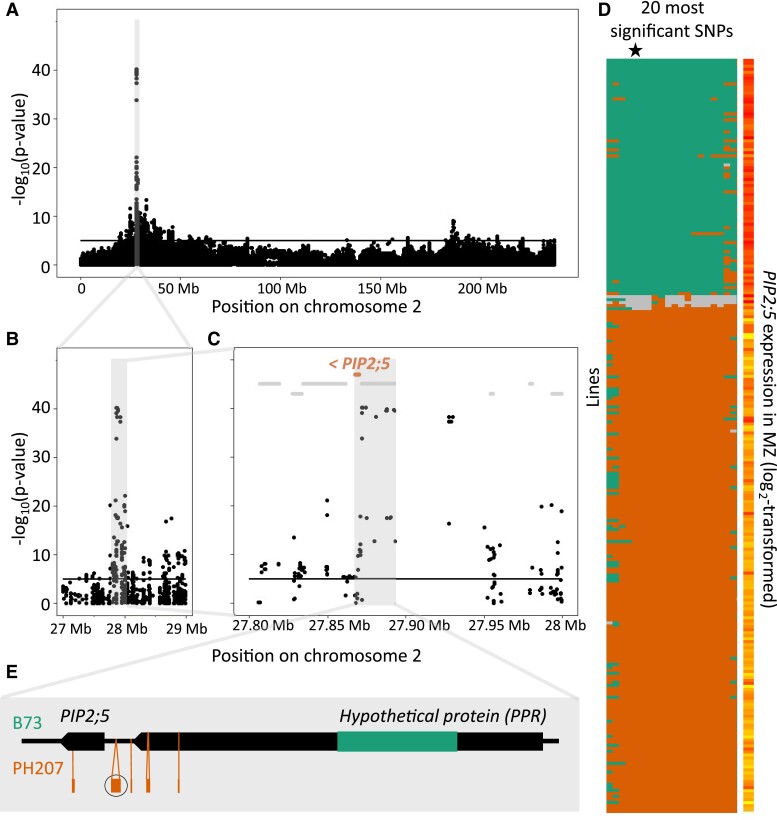
*PIP2;5* local eQTL. **A to C)** Manhattan plots showing the chromosome 2 **A)** and successive closer views of the local peak for *PIP2;5*_MZ (regardless of eQTL range definition) **B and C)**. Vertical axes represent the −log_10_(*P*-value). *PIP2;5* gene is highlighted in orange. Other genes are shown in gray. The black horizontal line represents the significance threshold of 5. **D)** Two haplotypes are defined by the alleles at the lead SNPs of the local eQTL. Lines are shown vertically, while the 20 most significant SNPs are displayed horizontally (labels are not visible due to the small scale), ordered according to their physical position (they cover a 235 kb range). The black star indicates the lead SNP (AX-91209412). Genotyping data are shown for the 20 most significant SNPs, colored in green for the B73 allele and orange for the other allele. The rare heterozygous alleles determined by the genotyping are colored in gray. *PIP2;5* expression data in the MZ are shown on the right, colored in a yellow (low) to red (high) gradient. **E)** Schematic representation of the locus containing *PIP2;5* and the next upstream annotated gene^4^, covering a region of ∼25 kb. B73- and PH207-specific large indel polymorphisms (>50 bp) are represented in green and orange depending on the lines they are present in, respectively. The PH207 indel circled in black encodes a MITE transposon. Arrowheads indicate the 5′-3′ gene orientation.

Both local *PIP2;5* eQTLs detected in EZ and MZ were considered as one, as their effects worked in the same direction and the lead SNPs showed close proximity (359 bp) and nearly identical allele segregation in the 252 lines of the panel. Therefore, the lead SNP of the *PIP2;5*_MZ data set (AX-91209412) was used as reference. Alleles at this position defined 2 distinct haplotypes in the population, extending over a large region (Fig. 6). Among available reference genomes at the time of the experiments (B73, PH207, and B104, matching the panel), B73 and PH207 were used as representatives of the first and second haplotypes, respectively. The B73 allele was associated with a positive effect on *PIP2;5* expression. Genomic sequence comparison showed a high identity of the *PIP2;5* coding sequence (98.72%). It is noteworthy that a point mutation close (7 bp upstream) to the 3′ splice site of the last intron was mapped and identified as putatively associated with splicing variation in the Ensembl Plants database. Collinearity between the genome sequences was observed up to ∼30 kb upstream of *PIP2;5*, allowing the sequence alignment and identification of multiple indels, but was lost afterward (Supplementary Fig. S4). A large (∼6 kb) indel polymorphism was present in the B73 sequence (absent from PH207), ∼11.5 kb upstream of *PIP2;5*, within the next upstream gene (GRMZM2G178681–Zm00001d003007) annotated as belonging to the pentatricopeptide repeat (PPR) superfamily protein (or in the intergenic region, depending on the reference genome versions). Several indels, closer to the *PIP2;5* gene, were present only in the PH207 sequence and absent from B73 (Fig. 6E). One indel was also identified in the *PIP2;5* gene itself, within the last intron (present in PH207, absent from B73).

The presence of a 457 bp indel located ∼430 bp upstream of the *PIP2;5* transcription starting site in PH207 was of particular interest. The major part of this indel (303 bp) was identified as a putative MITE transposon using a MITE-finder blast tool (highest score *E*-value = e^−166^; *P*-MITE; Chen et al. 2014). Blast results suggested that this MITE belongs to the maize *Heartbreaker* (*Hbr*) family, characterized by particular terminal inverted repeats (TIR), a conserved length (∼310 bp), and variable target site duplications (Zhang et al. 2000;Supplementary Fig. S5).

**Figure 7.**
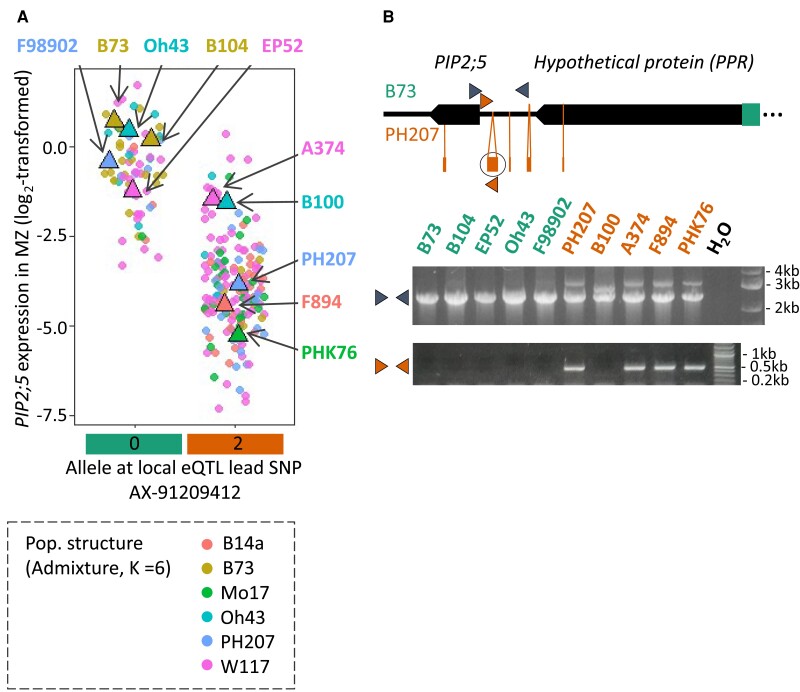
MITE-containing indel conservation. Five lines were selected among each subpopulation defined by the allele at the local eQTL to look for the conservation of the MITE-containing indel. **A)** Selected lines are highlighted on a graph representing *PIP2;5* expression on the MZ according to the allele at the lead SNP of the local eQTL. **B)** PCR amplifications using as template gDNA from the chosen hybrids (inbred line × UH007). Middle panel, amplification of the *PIP2;5* promoter region (primers shown by blue arrows—top panel) allowing the identification of 2 main alleles associated with the B73 and B104 (lower band) or PH207 (upper band) reference genomes, respectively. Lower panel, amplification figuring the presence of the MITE-containing indel (circled in black) thanks to both an internal and an external primer (shown by orange arrows).

The presence/absence of this particular MITE-containing indel was assessed in 10 lines (B73, Oh43, B104, F98902, and EP52 for the B73 haplotype and PH207, A374, B100, F894, and PHK76 for the PH207 haplotype) chosen for maximizing the admixture group diversity by specific amplifications of *PIP2;5* promoter region (Fig. 7). Bands of different sizes depending on the haplotypes were observed supporting a widespread conservation and a good correlation with the unfavorable, PH207-like, haplotype of the eQTL. Only B100 did not coincide, as it did not harbor this MITE insertion, but yet the second smaller, further upstream, indel included in the targeted promoter region, responsible for the intermediate size band in the upper panel. Sequencing revealed that the MITE-containing indel was perfectly identical in the PH207, A374, F894, and PHK76 genotypes. Table 4 gathers the conservation patterns for the polymorphisms judged as the most promising to explain this local *PIP2;5* eQTL, obtained from sequenced genomes or the manual sequencing from gDNA samples. We considered particularly the MITE-containing indel located the closest to *PIP2;5* and the larger ∼6 kb indel located further away. We also looked at the conservation of the indel located in the last *PIP2;5* intron as well as the putative splice site variant SNP mapped in Ensembl Plants. Altogether, the MITE-containing indel (except for B100) and the ∼6 kb indel (at least the ∼3.5 kb part of it that was conserved in Oh43 and UH007 as well) showed the best coincidence with the eQTL haplotype.

**Table 4.** Structural polymorphisms around *PIP2;5* gene

Accession	*PIP2;5* expression in MZ	Local eQTL lead SNP AX-91209412	MITE indel (457 bp)	Intron indel (235 bp)	Large indel (5,915 kb)	Splice variant (position: 2:27869387)	Source
PHK76	−5.31	0	+	+	−	+	Man
NC358	−5.16	0	+	−	−	+	Man
MS71	−4.66	0	+	+	−	+	Ref
F894	−4.39	0	+	+	−	+	Man
PH207	−3.89	0	+	+	−	+	Ref/man
Mo17	−3.51	0	+	+	−	+	Ref
B100	−1.59	0	−	−	−	+	Man
A374	−1.45	0	+	+	−	+	Man
B97	−1.20	1	+	+	−	+	Ref
EP52	−1.26	2	−	+	+	+	Man
F98902	−0.39	2	−	−	+	−	Man
B104	0.24	2	−	−	+	−	Ref/man
Oh43	0.43	2	−	−	Partial	+	Ref/man
B73	0.70	2	−	−	+	−	Ref
UH007	…	2	−	−	Partial	…	Man

Alleles at polymorphisms are indicated for 14 different lines of the panel and the common parent UH007 as well. Plus (+) means “presence,” while minus (−) means “absence.” Ellipsis indicates the absence of information. Source indicates whether the information comes from the sequenced genomes (ref) or the manual sequencing (man) from cDNA samples (hybrids). *PIP2;5* relative expression in the MZ is shown, and accessions have been ordered following their *PIP2;5* expression level in MZ.

### MITE-containing indel affects the promoter activity

**Figure 8.**
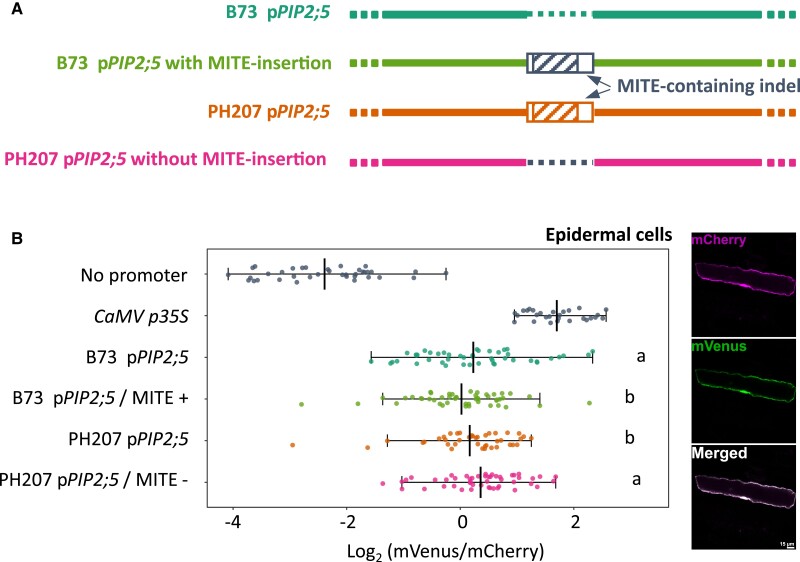
MITE-containing indel effect on the *PIP2;5* promoter activity. **A)** Constructs used (not drawn to scale). The green and orange/magenta backbones figure the B73 and PH207 *PIP2;5* promoter sequences. The rectangle indicates the presence of the MITE-containing indel (the hatched part representing the MITE itself), while the dashed line indicates its absence. Dark blue features indicate the artificial constructions. **B)** mVenus and mCherry fluorescent signal ratio in the leaf epidermal cells. The *mVenus* expression was driven by the tested promoter constructs as indicated in the vertical axis labels, while *mCherry* expression was driven by the double minimal *CaMV p35S* promoter, carried by the same vector and used as normalizer. Results are presented as log_2_(mVenus/mCherry) and group 3 independent experiment repetitions performed blind. Each dot represents an individual cell. Statistical tests were performed with a linear mixed model testing the background effect (B73 or PH207), the MITE-containing indel effect (presence or absence, referred to as “MITE+” and “MITE−,” respectively, in the axis labels), and their interaction, with a random intercept taking the experience replication dependency into account (SAS or nlme function in R). Significance threshold was set to *P* < 0.05. Representative pictures of transformed epidermal were added, channels (top panel, mCherry; middle panel, mVenus; lower panel, both merged). The scale bar represents 15 *µ*m.

The MITE-containing indel stood as a promising causal candidate for the local eQTL, due to its strategic position in the promoter and the recurrence of demonstrated MITE-associated QTLs (Magalhaes et al. 2007; Salvi et al. 2007; Hou et al. 2012; Zerjal et al. 2012; Castelletti et al. 2014; Mao et al. 2015). In order to demonstrate its effect on the promoter activity, we compared the *PIP2;5* promoter (*pPIP2;*5) activity of both B73 and PH207 using a dual fluorescence reporter assay. The tested promoter sequence was defined from *PIP2;5* start codon (nonincluded) up to the next upstream annotated gene, meaning a 1,884 and 2,600 bp region for B73 and PH207, respectively (similar to the blue arrows-targeted region in Fig. 8). Promoter activity was monitored with mVenus (*pPIP2;5*:*mVenus*) as a reporter, mCherry (*CaMV p35S*:*mCherry*) being used as a normalizer (polycistronic vectors). Both fluorescence intensities were recorded from confocal microscopy images coming from maize leaf cells transiently transformed by biolistic particle delivery.

In order to remain as close as possible to the physiological reality, and because *PIP* gene expression is known to be sensitive to environmental conditions (diurnal cycle, light, humidity, etc.), the entire plants were used for the bombardment and kept complete and alive in their usual growing conditions until the signal observation (∼40 h), unlike usual protocol using cut leaf pieces conserved on solid media in the dark (Chevalier et al. 2014). Thus, for practical reasons, only young plants were used for transformation (∼12 d, usually 4th fully emerged leaf). Demonstration of the MITE-containing polymorphism effect was carried out by synthetically removing and adding the whole MITE-containing indel from PH207 and to B73 *PIP2;5* promoters, respectively, by triple PCR. This approach allowed distinguishing the effect of the “background promoting sequence” (B73 or PH207) and the MITE-containing indel itself (presence or absence; Fig. 8). We observed that the Venus/mCherry ratio was lower when the MITE-containing indel sequence was present (either inserted in *pPIP2;5* from B73 [B73 p*PIP2;5* MITE+] or naturally present in PH207 p*PIP2;5*) compared with the *pPIP2;5* without the MITE-containing indel sequence from both backgrounds (Fig. 8B). Statistical analysis revealed the significance of the MITE-containing indel (*P* = 0.0271*) but not the background sequence on the measured promoter activity. The interaction of MITE-containing indel presence and background sequence was tested as nonsignificant. This demonstrated the effectiveness of the MITE-containing indel on the *PIP2;5* promoter activity, independently of the background sequence, and the effect (without MITE-containing indel > with MITE-containing indel) agrees with the eQTL effect (B73 > PH207).

## Discussion

In this work, we investigated the expression diversity of 4 *PIP* aquaporin genes at a large scale and mapped numerous eQTLs, both local and distant. They constitute valuable starting points for the identification and characterization of potential regulatory elements and factors for *PIP* gene expression, illustrated here by the identification of a MITE transposon polymorphism in the *PIP2;5* promoter.

Maize is ideally suited for GWAS, thanks to its important genetic diversity and rapid LD decay (Buckler et al. 2006). However, the high significance of some local eQTLs was difficult to handle as it spread rapidly over neighboring regions as soon as some genetic linkage was still present. In some cases, we observed a large zone of pyramiding eQTLs spreading over a large portion of the chromosome. Since the strongest associated SNP explained a large part of the variation of *PIP* expression (Table 1), most additional local eQTLs probably originated from SNPs that were in LD with the main causal polymorphism. However, these eQTLs blurred the detection of some other local eQTLs that were linked to other causal polymorphisms. How to distinguish local eQTLs that capture different causal polymorphisms acting independently on gene expression and how to merge eQTLs that are in LD with each other were important questions. Indeed, the genetic determinism of the observed gene expression variation was likely associated with far fewer loci than implied by this high number of eQTLs, so it was necessary, for biological and practical reasons, to disentangle those problematic regions. Therefore, to handle those “local eQTL flooding zones,” we implemented a multilocus model the so-called covariate approach (Segura et al. 2012). It allowed us to remove eQTLs that captured the same causal polymorphisms and bring to light new ones that are independent from the first one. Since genetic determinism of gene expression is likely oligogenic, we believed that the covariate approach may be valuable in larger-scale studies as well to point, for instance, at multiple local eQTLs, even if more stringent eQTL selection criteria are applied.

### Variability of *PIP* gene regulation

The diversity panel and the eQTL mapping methodology applied in this work revealed that (i) a significant variability in *PIP* gene expression exists, even across economically and agronomically relevant elite lines, and (ii) this variability is primarily associated with local eQTLs in the observed conditions. Interestingly, the patterns of variation were different among the studied isoforms: most of them showed a rather homogeneous variation irrespective of the population structure, while *PIP2;5* showed a clear interheterotic group variation (B73 and Oh43 > PH207 and B14a and Mo17). This major difference in expression, whose important genotypic foundation has led to the mapping of the cognate major local eQTL, was likely the result of an unbalanced allelic composition at the *PIP2;5* locus and its surroundings in the different heterotic groups. While this should be investigated further, this distinction is a clear heritage of past breeding, which would likely be confirmed by the investigation of specific selection signs in the SNP composition in this region. Whether *PIP2;5* expression (and any associated phenotype of interest) was the intended target of this past selection or, on the contrary, a side effect of a selection affecting a closely related locus is unclear so far. The investigation of these particular heterotic groups and their potentially contrasting phenotypes such as tolerance to specific climatic scenarios or hydraulic parameters might help answer this question. Regarding the prevalence of local eQTLs, specific functional characterization would be necessary to confirm the *cis* nature of the underlying regulatory mechanisms. The observation of 2 different leaf zones pointed to the different nature of most distant eQTLs, which could point to zone-specific regulation mechanisms. The consistency of the multiple local eQTLs identified for a unique isoform (2 zones and secondary local eQTLs identified by covariate GWAS) should be assessed more carefully by looking at local LD patterns and haplotypes among the population.

*PIP1;1* expression varied in lower magnitude than other genes in the studied diversity panel. Such an implicit low variation in expression was interesting from a biological point of view. *PIP1;1* is a ubiquitous PIP isoform, expressed at high levels both in roots and aerial parts. Mechanistically, its channel properties are not yet resolved. Its permeability to water is ambiguous (Fetter et al. 2004). The difficulty of obtaining *pip1;1* knockout lines also supports its importance as an essential gene. So far, only partially silenced lines showing reduced but not null expression could be obtained (Ding and Chaumont, personal communication). The stable expression observed, here, across the diversity panel and the subsequent rather weak eQTLs detected are yet other arguments supporting the exceptional character of *PIP1;1* among the PIP subfamily. These observations may suggest a fundamental role for this specific isoform compared with a more adaptive role for the other isoforms, compatible with a higher degree of freedom and proneness to variations. A deeper investigation of the local and distant eQTLs reported in this work and other studies may bring valuable information to support or overturn this hypothesis.

In the future, effort should be made to benefit from, and connect, the different data collected for the same DROPS panel (Millet et al. 2016; Prado et al. 2018; Negro et al. 2019). In particular, phenotypical data related mainly to plant hydraulic parameters are available for the experiment from which our tissue samples were directly collected (Prado et al. 2018). Integrating all of them will be an opportunity to assess the involvement of *PIP* (and other aquaporin) genes on physiological and agronomical traits. The comparison of the (e)QTLs from our work and a hydraulic-oriented sister study (Prado et al. 2018) only revealed a few colocalizations, but they would still deserve closer attention. The absence of colocalization at the local *PIP* eQTL suggests that, if aquaporins are important in these processes, their involvement is probably more subtle and coordinated within the whole family rather than relying on 1 major isoform and a putatively nonadjustable local eQTL *cis*-acting underlying regulation.

The final eQTL list was compared with other eQTL studies based on whole-transcriptome RNA sequencing data (Li et al. 2013; Liu et al. 2017; Wang et al. 2018; Pang et al. 2019). It is noteworthy that they concern other types of samples: kernels (Liu et al. 2017; Pang et al. 2019), shoot apex on 14-d seedlings (Li et al. 2013), and aerial part of 11-d-old seedlings (Wang et al. 2018). They also worked on other diversity panels, including a maize–teosinte RIL population in the case of Wang et al. (2018). Therefore, it represented an opportunity to identify eQTLs that may be conserved across tissues and developmental stages and others that may be more specific to the particular physiological conditions. In addition, each study applied its own criteria, more or less stringent, for eQTL definition, characterization (local vs. distant), and selection. Supplementary Fig. S6 gathers the results from this work and these other studies. All local eQTLs were supported by several or all studies (in case of *PIP2;5* and *PIP1;3*). Colocalizations between distant eQTLs were detected as well, namely, for a *PIP1;1*_EZ eQTL (QTL_3) colocalizing with a *PIP1;1* distant eQTL from Liu et al. (2017) (for kernel tissues) and a *PIP1;3_*EZ (QTL_9, covariate GWAS) distant eQTL colocalizing with a distant *PIP1;3* eQTL from Pang et al. (2019) (for kernel tissues). In both cases, they are located quite close to the local loci.

### A MITE transposon regulates *PIP2;5* expression

A very significant eQTL peak located on the *PIP2;5* gene, both in leaf MZ and EZ. This large peak was composed of multiple pyramiding eQTLs. Patterns of local LD between SNPs from these different eQTLs suggested that these multiple eQTLs captured the same local causal polymorphisms regarding the large effect of main local eQTL (16% and 50% of the total variance of *PIP2;5* expression in EZ and MZ leaf zone, respectively [Table 3]). This was further confirmed by the covariate GWAS since the integration of local eQTL lead SNP wiped out most neighboring ones. As a whole, data suggest the presence of a sole local eQTL for *PIP2;5*.

Significant SNP segregation profiles and sequence comparison between lines diverging at the local lead SNP, B73, and PH207 allowed identifying 2 rather long haplotypes. Among the different polymorphisms identified, a MITE-containing indel, present in PH207 and located at ∼430 bp from the start codon, stood out as a promising causative candidate, because of (i) its good apparent coincidence with the eQTL assessed on 14 lines, (ii) its strategic position in the promoter, and (iii) the data accumulating on MITEs, highlighting their role as genomic, genetic, and phenotypic diversity drivers. The MITE located in the PH207 *PIP2;5* promoter shares the features of the maize *Hbr* family (included in the *tourist* superfamily): conserved TIR, similar length (∼310 bp), and diverse target site duplication. This family was reported to show a high degree of sequence conservation (usually ∼90% sequence identity) suggesting their late origin and recent or current spreading activity (Zhang et al. 2000). Indeed, in rice, 3 elements (mPing, mGing, and mJing) belonging to different families of *tourist* MITE, with variable intrafamilial sequence similarity degrees—reflecting multiple historical amplification bursts (Lu et al. 2012)—were shown to still be active (Jiang et al. 2003; Naito et al. 2006; Dong et al. 2012; Tang et al. 2019). However, the lower sequence similarity of the identified MITE with the few other *Hbr* that were specifically observed, as well as the polymorphisms observed in one of the TIR, probably suggests a relatively ancient origin and lost transposition activity. In any case, *Hbr* MITEs were proposed as valuable molecular markers for genotyping and genetic relationship prediction because of their stability, high degree of—presence/absence—polymorphism, and preference for genic regions (Casa et al. 2000, 2002, 2004; Zerjal et al. 2012; Venkatesh 2020). Therefore, like other transposons, but even more because of their smaller size and high copy number, MITEs shape genomes and create genomic diversity. They were proposed to have a major role in the formation of clusters, gathering related biosynthetic genes, for instance (Boutanaev and Osbourn 2018). However, beyond the genome structure, their integration and polymorphisms may potentially have consequences at the genetic, epigenetic, and phenotypic levels.

Genome-wide studies on 2 rice cultivars showed that genes with MITE insertions within the 500 bp upstream or downstream regions, or within introns, have significantly lower expression than genes located away from MITEs (Lu et al. 2012); however, no obvious effects of MITE insertions on individual gene expression were emphasized when crosschecking presence/absence variations and expression levels between cultivars in the same study. While most insertions are probably neutral (or have still undetected effects; Naito et al. 2009; Lu et al. 2012), in some cases, the local influence of MITEs on gene expression was concrete and attributed to several nonexclusive mechanisms. MITEs can modulate the nearby gene expression through the introduction of regulatory motifs, with positive, negative, or condition-specific effects (Yang et al. 2005; Naito et al. 2009), or via MITE-derived small RNAs (Kuang et al. 2009; Wei et al. 2014; Xin et al. 2019). MITE impacts on gene regulation may also come from RNA-independent DNA methylation (Yang et al. 2005), gene product modifications or truncations due to—yet rare—MITE insertion into exons (Guillet-Claude et al. 2004), or splicing alteration due to MITE insertion into introns (Balzan et al. 2018; Xin et al. 2019). Therefore, MITEs may have consequences at the phenotype level (Bureau and Wessler 1992; Tang et al. 2019, for instance) and were repeatedly proposed as the causal variations underlying QTLs for agronomic traits and/or historical evolution understanding. A MITE insertion into a peroxidase gene involved in lignification presumably explains a major QTL for maize cell wall digestibility, due to gene product truncation (Guillet-Claude et al. 2004). A sorghum aluminum tolerance QTL is also likely due to a MITE insertion upstream of a multidrug and toxic compound extrusion protein *MATE* gene, but the (de)regulatory mechanisms have not been characterized (Magalhaes et al. 2007). MITE insertions are also associated with several flowering-time genes and QTLs in maize, sorghum, and rapeseed (Salvi et al. 2007; Hou et al. 2012; Zerjal et al. 2012; Castelletti et al. 2014). The major flowering-time QTL *Vgt1* could be at least partly attributed to stable methylation spreading from a MITE, downregulating *ZmRap2.7* flowering-time gene transcription (located 70 kb downstream) in maize and sorghum (Salvi et al. 2002, 2007; Castelletti et al. 2014). The involvement of this particular locus was also supported by an open chromatin state coinciding with the MITE (Rodgers-Melnick et al. 2016) and long-range chromatin interaction with a nearby sequence (Peng et al. 2019), thus suggesting maybe the coincidence of multiple regulatory mechanisms. In addition, a *tourist* MITE (located ∼60 kb from *TB1*) underlying the major QTL for apical dominance was shown to act as a repressor by dual luciferase assay. The QTL causative variation was, however, attributed to the close *Hopscotch* retrotransposon whose positive regulatory effect was consistent with the evolutionary data (Studer et al. 2011). Here again, it was further supported by genome-wide structural chromatin assay and long-range interaction detection (Rodgers-Melnick et al. 2016; Li et al. 2019). Finally, the cloning of a maize drought tolerance QTL revealed that a MITE inserted in the promoter of a *NAC* gene (*ZmNAC111*) reduced its expression via RNA-dependent DNA and histone methylation, thus lowering seedling drought tolerance (Mao et al. 2015). This particular MITE was particularly present in the temperate maize germplasm (Mao et al. 2015).

In our case, the negative effect of the MITE-containing indel on the *PIP2;5* promoter activity was demonstrated by a confocal microscopy–based reporter assay. The exact MITE-containing indel effect was highlighted by artificial insertion/deletion of the whole indel in and from the B73 and PH207 promoter sequences, respectively. Unfortunately, scales of such different experiments (reporter assay and RT-qPCR) did not easily match, so the magnitude of the effect relative to the expression could not be quantitatively estimated. Whether the observed effect was due to the MITE itself or the non-MITE portion of the indel was not determined. *Cis*-regulatory elements (including putative NAC, MYB, LAE, and other binding sites) were identified within the whole indel (including within the MITE) using the PlantPAN 2.0 TFBS promoter scanning tool (Chow et al. 2016) so that it might allow/increase the binding by *trans*-regulatory factors (see Supplementary Fig. S7). In addition, the MITE could be subjected to RNA-dependent DNA methylation, as small RNA database screening suggests its specific targeting by siRNAs (Supplementary Fig. S8, Johnson et al. 2007). Silencing methylation could then potentially spread across the *PIP2;5* promoter and gene region. However, mining available methylation profiles showed no particular difference in methylation at the *PIP2;5* gene and promoter location when comparing lines matching the panel and differing at this MITE-containing indel (including B73, B97, Mo17, MS71, and Oh43; Eichten et al. 2013), reducing the likelihood of this hypothesis.

In addition to the MITE-containing indel, other regulatory elements might also contribute to this very significant *PIP2;5* local eQTL. The compelling uncovering of the local regulatory elements could be achieved with a fine mapping in specifically bred nearly isogenic or backcrossed populations and by comparing and dissecting the effect of the local haplotypes in ideally identical genetic backgrounds. Another possibility of narrowing or defining the eQTLs lies in the integration of polymorphic indels of various sizes that have been genotyped in a large diversity panel including the one used in this work (Mabire et al. 2019).

In conclusion, such work on the dissection of the *PIP* gene expression variability is a valuable approach to ultimately elucidate the networks involved in the crop water homeostasis and their essential adaptation to various and ever-changing environmental scenarios.

## Materials and methods

### Plant material

The diversity panel was generated by crossing a common maize (*Z. mays*) flint parent (UH007) with dent lines having a restricted flowering-time variation (European DROPS project). The resulting panel comprised 252 hybrids with a 7-d flowering window and was used in several previously published studies (Millet et al. 2016; Prado et al. 2018; Negro et al. 2019; Blein-Nicolas et al. 2020). The panel was then divided into 6 genetic groups identified using the ADMIXTURE software (Negro et al. 2019). These groups were defined as follows: stiff stalk containing 32 lines, iodent containing 34 lines, Lancaster with 32 lines, Lancaster early containing 16 lines, stiff stalk early with 36 lines, and, finally, W117 admixture, which includes 102 lines that did not fit into the other groups, such as W177, D06, and NC358. One line of each group was used as a reference (B73, PH207, Mo17, Oh43, B14a, and W117 lines for stiff stalk, iodent, Lancaster, Lancaster early, stiff stalk early, and W117 admixture, respectively).

In our case, the panel was grown in May 2013 in the phenotyping platform PhenoArch (LEPSE, INRAE, Montpellier) under semicontrolled conditions. Well-watered condition (soil water potential of −0.05 MPa, retention capacity) was maintained by automatic and personalized watering of the pots 3 times per day. The EZ samples (yellow cylinder at the stem base, covering green sheath removed) were collected after 10 d of culture, at 4-leaf stage, while the MZ samples (middle of the expanded leaf blade) were collected after 44 d, at 14-leaf stage, on leaf 10. Three successive days (3 h timeframe in the morning: 7:30 to 10:30 Am) was required to collect all samples while limiting the sampling time variation. Three biological replicates coming from different pots were harvested for each genotype. Samples were harvested and immediately frozen in liquid nitrogen and kept at −80 °C until RNA extraction.

For biolistic transient expression, B104 inbred seeds were surface disinfected with 50% (v/v) sodium hypochlorite for 5 min and rinsed 6 times with distilled water. Seeds were placed vertically between wet tissue papers in the dark for 2 d at 30 °C and transferred into soil in individual 6 cm diameter peat Jiffy pots filled with 80% (v/v) classic potting soil (DCM) and 20% (v/v) vermiculite (Agra-vermiculite) and grown under an 8/16 h dark/light regime (18 °C/25 °C; semicontrolled growth chamber conditions).

### RNA extraction

Frozen tissues were ground with metallic beads using a Mixer Mill MM 400 grinder (Retsch), and RNA extraction was performed with the Spectrum Plant Total RNA Kit (Sigma-Aldrich). On-column DNA digestion was performed with DNAse I (Sigma-Aldrich) as recommended. Complementary DNA (cDNA) synthesis was performed from 2 *μ*g of total RNA extract (measured with a Nanodrop ND-1000 [Isogen]), with M-MLV reverse transcriptase (Promega) in the presence of RNasin (Promega), according to the manufacturer's instructions. cDNAs were then purified with the NucleoSpin PCR Clean-up kit (Macherey-Nagel) and eluted in 50 *μ*L of preheated (65 °C) sterile water.

### RT-qPCR

The expression of 4 *PIP* genes (*PIP1;1*, Zm00001d002690; *PIP1;3*, Zm00001d051403; *PIP2;2*, Zm00001d005421; and *PIP2;5*, Zm00001d003006) was measured for the whole panel on the leaf EZ and MZ by RT-qPCR. RT-qPCR was carried out with primers (Supplementary Table S1) according to the protocol already described in Hachez et al. (2006) and Heinen et al. (2014), using the SYBR Green Master Mix 2× (Eurogentec) and StepOnePlus Real-Time PCR system (Applied Biosystems). They were performed in 96-well plates designed in a gene-optimized way: the 7 genes (4 *PIP* genes + 3 reference genes) were measured in a same plate for the 3 biological replicates of a same genotype (with 2 technical replicates). The results were normalized via the 2ΔCt method (Ct for cycle threshold) against the 3 reference genes (*Act1*, Zm00001d010159; *EF1α*, Zm00001d046449; and *Ubi*, Zm00001d053838), leading to relative expression data. The relative expression data were used as log_2_ transformed in all subsequent analyses. Analysis of the putative polymorphisms within the primer sequences was described in Supplementary Appendix S1.

For each trait (gene expression in a given zone), the linear mixed model was fitted:


Y=G+E

where *Y* is the vector of phenotypic observation, *G* is the random genotypic effect, and *E* is the residual effect. The heritability was then computed as *V*_g_/(*V*_g_ + *V*_e_/*n*) where *V*_g_ is the variance attributed to the genotype, *V*_e_ is the residual variance, and *n* is the number of replicates.

### GWAS

Lines were genotyped using 3 genotyping technologies: an Illumina HD 50K array developed by Ganal et al. (2011), an Affymetrix Axiom 600K array (Unterseer et al. 2014), and genotyping-by-sequencing, as described by Negro et al. (2019). The resulting collection gathered more than 960,000 SNPs, among which 737,202 with a minor allele frequency (MAF) > 0.05 were used for the GWAS. GWAS was implemented as described by Millet et al. (2016), Prado et al. (2018), and Negro et al. (2019). In brief, GWAS was performed on each trait (combination *PIP* gene × leaf zone, referred to as *PIPX;X*_MZ/EZ) individually, using the following single locus mixed model: *Y* = *µ* + *Xβ* + *G* + *E*, where *Y* is the vector of phenotypic values, *µ* is the overall mean, *X* is the vector of SNP scores (the genotype value at the tested marker for all the lines), *β* is the additive effect of the tested marker (i.e. what we are testing), and *G* and *E* represent polygenic (approximately population structure effect) and residual effects, respectively. *G* modulates the genetic pairwise relatedness (or kinship) between individuals and was estimated from the 50K marker collection (Negro et al. 2019). In practice, it was derived from all SNPs except for those on the chromosome containing the marker being tested, according to the procedure of Rincent et al. (2014b). The marker effect *β* was estimated for each SNP by generalized least squares, and their significance was tested with an *F*-statistic. The −log_10_ of the *P*-values was retrieved and significance threshold was set to 5 as detailed in Negro et al. (2019). Analyses were performed with FaST-LMM v2.07 (Lippert et al. 2011). Inputted trait data were genotypic means (means of the 3 biological replicates), as log_2_-transformed relative expression data.

The grouping of the significant SNPs into QTLs and, hence, the eQTL delimitation was based on estimation of the LD windows for each SNP (LD_*win* approach; Negro et al. 2019). Significant SNPs with overlapping estimated LD windows (threshold of LD corrected for cryptic relatedness *r*^2^*K* = 0.1) were considered as belonging to a single QTL. QTL intervals were delimited by the external limit of the LD window of the most extreme SNPs. Once collected and delineated, QTLs were defined by the characteristics of their lead SNP (= the most significant SNP). Colocations of QTLs from different analyses were defined by QTL interval overlapping.

Conditional GWAS were performed by introducing a SNP of interest as covariate in the GWAS model. It was generally implemented to integrate the powerful local eQTLs (defined as including the cognate *PIP* gene—delineated as the transcribed region, according to the B73 reference genome version 4 annotation—within its range), thus removing all redundant signals and shedding light on putatively hidden eQTLs of lower effects. It was also used to integrate a putative confounding effect arising from a reference gene used for the RT-qPCR, after the discovery of a QTL located on this gene (*Ubi*) for the *PIP1;1_EZ* data set. This so-called *ubi*-adjustment was implemented for both *PIP1;1* data sets.

### Genetic constructs for promoter activity evaluation

All constructs used for transient expression were derived from the p*EGB 35S:Luciferase:Tnos* plasmid (GB0110, Addgene plasmid no. 68216; Sarrion-Perdigones et al. 2013). A USER cassette was introduced by restriction and ligation upstream of the *Luc* coding sequence between *Nco*I and *Sfo*I sites resulting in a plasmid named GB0110m. The latter was then used as backbone to swap the *Luc* gene with a *mVenus* reporter coding sequence, to be able to observe and quantify the promoter activity by microscopy. This was obtained by digesting GB0110m with *Pac*I and *Afl*II and inserting the *mVenus* coding sequence (amplified and adapted for the insertion with 2 pairs of primers used successively, venus_GB110_F1-R1/F2-R2; Supplementary Table S5). The plasmid was named GB0110m-mVenus. In order to have a transfection control within the vector, a double minimal cauliflower mosaic virus *35S* promoter *CaMV p35S*:*mCherry*:*tNOS* cassette was inserted at the *Aat*II restriction site (K7mCherry_AatII_F/R primers). The ultimate vector was named GB0110m-mVenus-K7mCherry.

### MITE-containing indel detection and *PIP2;5* promoter amplification

The presence/absence of the MITE-containing indel into the *PIP2;5* promoter region of specific lines was assessed with different primer pairs (Supplementary Table S5; Fig. 8). The *PIP2;5* promoter region (∼2 kb, up to the next upstream annotated gene) and the MITE sequence amplification resulted in either bands with different sizes or the presence/absence of the indel, respectively.

Both *PIP2;5* promoter versions were amplified by PCR from B73 and PH207 pure lines and cloned into pGEM-T Easy vector (Promega) for sequence validation. The promoter region was selected from the limit of the first upstream annotated gene (including a part of the presumed 3′ UTR) up to *PIP2;5* translation initiation ATG (not included), including the 5′ UTR, consisting of a 1,884 and 2,600 bp region for B73 and PH207, respectively. Validated promoter regions were then inserted into GB0110m_mVenus_K7mCherry in the USER cassette, with the appropriate cloning procedure (Nour-Eldin et al. 2006; F_user_GB0110m_prom1 and R_user_5UTR_G0110m_prom). To demonstrate the role of the MITE-containing indel present in the PH207 *PIP2;5* promoter allele, versions of the B73 and PH207 *PIP2;5* promoters were created by adding or removing, respectively, the whole MITE-containing indel sequence by triple PCR (primers in Supplementary Table S5).

### Biolistic transient transfection of leaf epidermal cells

Bombardment of leaf tissues with plasmid DNA–coated microparticles was performed using the Biolistic PDS-1000/He Particle Delivery System (BioRad) according to the manufacturer's instructions and following a protocol adapted from Chevalier et al. (2014). For each bombardment, 0.5 mg of 0.6 *µ*m gold microbeads (BioRad) was used, coated with ∼2 *µ*g of plasmid DNA. Twelve-day-old plants (B104 inbred line) were used for transfection. They were removed from soil, and roots were washed for practical purpose. The median portion of the last fully developed leaf (usually the 4th leaf) was attached on a Petri dish lid, adaxial face upward, and placed on the target shelf of the bombardment chamber, 3 cm below the macrocarrier launch assembly. The whole plant stood in the lower part of the chamber. The macrocarrier flight distance was set to 11 mm, while the gap distance was set to 10 mm. Vacuum was applied up to 29″ Hg (98 kPa) before firing (1,100 psi rupture disk—7,600 kPa). Plants were then carefully transferred back into soil, adding stakes if necessary, to keep them in the best possible shape. Plants were kept in the usual dark/light conditions for ∼40 h before observation by microscopy.

Leaf epidermal cells were screened based on the transformation control (mCherry) and were imaged using a Zeiss LSM710 confocal microscope equipped with a spectral detector (C-Apochromat 40×/1.20 aqueous immersion objective). mCherry and mVenus were excited using 561 and 514 nm laser track, respectively. Emitted light was collected through a dichroic mirror on detectors 583 to 650 nm (561/633 MBS; master gain 700) for mCherry and 519 to 564 nm (485/514 MBS; master gain 799) for mVenus. Settings were kept identical along all experiments. Images were analyzed with the FIJI software (Schindelin et al. 2012). Signal was first smoothed, and then a rectangle was drawn to frame the epidermal cell of interest as closely as possible. Selected region was manually adjusted to exclude signal coming from potentially adjacent transformed cells and oversaturated nucleus region when present. The mVenus/mCherry ratio was calculated based on the total fluorescence obtained within the defined mask for each marker. Statistics were performed using SAS, fitting a linear mixed model, with a random intercept taking the experience dependency into account. The ratiometric data were log-transformed to fulfill the assumptions. Bombardment, image acquisition, and analysis were performed blind for 3 repetitions.

### Accession numbers

Sequence data from this article can be found in the maize genome database (https://www.maizegdb.org/) under accession numbers Zm00001d010159 (*Act1*), Zm00001d046449 (*EF1α*), Zm00001d053838 (*Ubi2*), Zm00001d002690 (*PIP1;1*), Zm00001d051403 (*PIP1;3*), Zm00001d005421 (*PIP2;2*), and Zm00001d003006 (*PIP2;5*). Data regarding the primer sequences are presented in Supplementary Tables S1 and S5.

## Supplementary Material

kiae326_Supplementary_Data

## Data Availability

The data underlying this article are available in the article and in its online supplementary material.

## Supplementary data

The following materials are available in the online version of this article.

**Supplementary Figure S1.** Trait correlation.

**Supplementary Figure S2.** *PIP* gene expression data.

**Supplementary Figure S3.** Endogenous control gene location checking.

**Supplementary Figure S4.** B73–PH207 genome collinearity upstream of *PIP2;5.*

**Supplementary Figure S5.** The indel present in PH207 ∼430 bp upstream of the *PIP2;5* start codon contains a MITE.

**Supplementary Figure S6.** *PIP* gene eQTLs.

**Supplementary Figure S7.** Predicted transcription factor binding sites within the MITE-containing indel sequence.

**Supplementary Figure S8.** Small RNA aligning on the MITE-containing indel sequence.

**Supplementary Table S1.** List of primers used for the measurement of relative expression of 5 PIP genes by RT-qPCR.

**Supplementary Table S2.** Initial eQTLs.

**Supplementary Table S3.** eQTLs detected with all models.

**Supplementary Table S4.** Summary of eQTLs detected with all models.

**Supplementary Table S5.** Primers used for molecular cloning.

**Supplementary Appendix S1.** Analysis of the putative polymorphisms within the primer sequences.

## Dive Curated Terms

The following phenotypic, genotypic, and functional terms are of significance to the work described in this paper:

Act1 Gramene: Zm00001d010159
Act1 Araport: Zm00001d010159

PIP2;5 Gramene: Zm00001d003006
PIP2;5 Araport: Zm00001d003006

TFBS Gramene: Transcription Factor Binding Sites (TFBSs)
TFBS Araport: Transcription Factor Binding Sites (TFBSs)

PIP1;1 Gramene: Zm00001d002690
PIP1;1 Araport: Zm00001d002690

PIP1;3 Gramene: Zm00001d051403
PIP1;3 Araport: Zm00001d051403

PIP2;2 Gramene: Zm00001d005421
PIP2;2 Araport: Zm00001d005421

EF1alpha Gramene: Zm00001d046449
EF1alpha Araport: Zm00001d046449

Ubi2 Gramene: Zm00001d053838
Ubi2 Araport: Zm00001d053838
