# Supplementary material for: Genetic variability of aquaporin expression in maize: From eQTLs to a MITE insertion regulating PIP2;5 expression
Source: Plant Physiol. 2024 Jun 5;196(1):368–84. doi: 10.1093/plphys/kiae326 (PMC11376376; doi:10.1093/plphys/kiae326)
Supplement: kiae326_Supplementary_Data [file kiae326_supplementary_data.zip › SuppFiguresLegends 20240527.pdf]

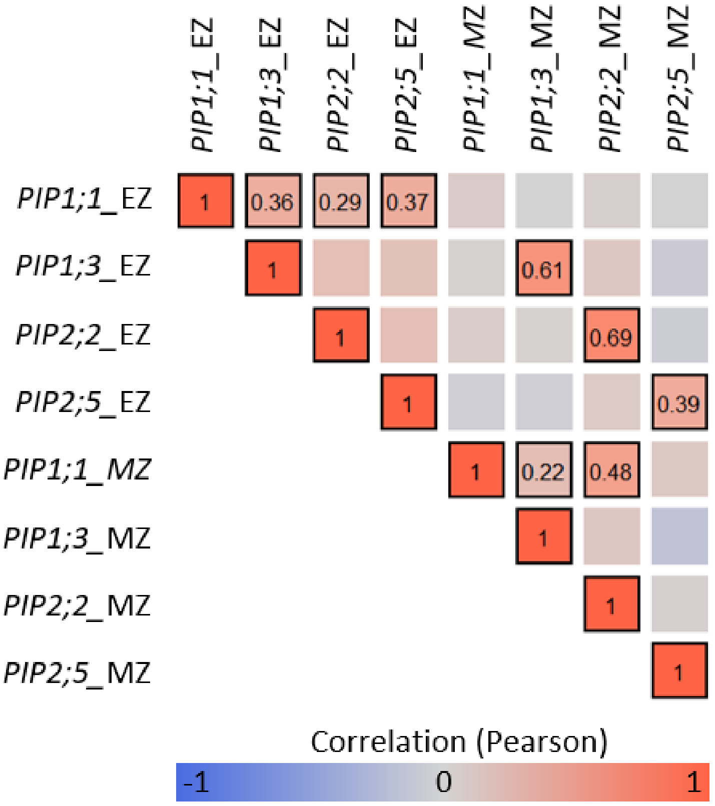

Supplementary Figure S1: Trait correlation. Matrix of correlation (Pearson) between *PIP* expression data in both EZ and MZ. Correlations are colored in blue (negative) to red (positive) gradient. Black frames indicate significant correlations (p-value < 0.05 with Holm correction for multitesting). Pearson's correlation factors and adjusted p-values were obtained with the rcorr.adjust R function (RcmdrMisc package).

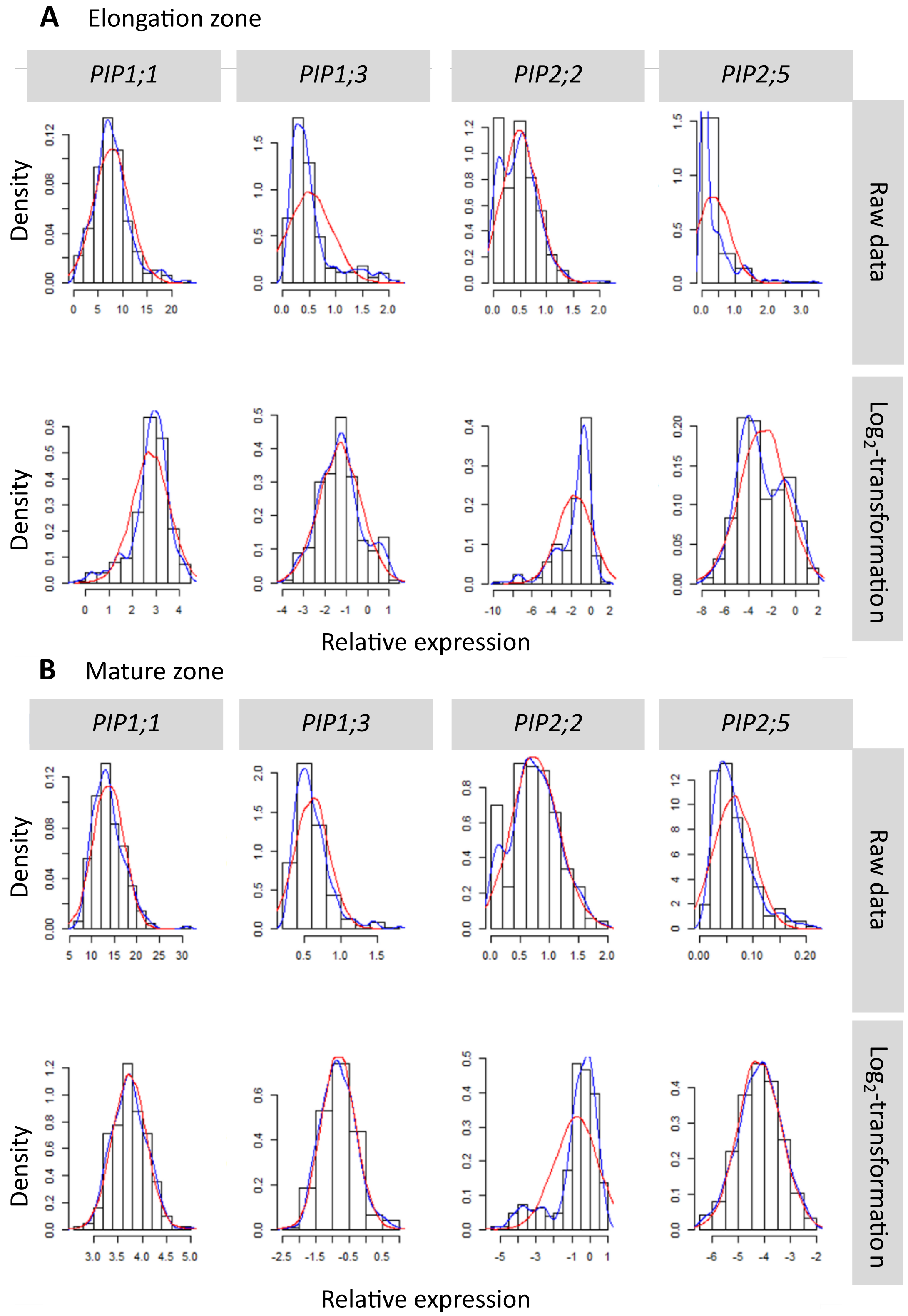

Supplementary Figure S2: PIP gene expression data. Distribution of the relative expression data for the four studied PIP isoforms in the elongation (A) or mature zone (B), shown either as raw data (upper panels) or after log2-transformation (lower panels). Blue lines are the actual density of the data and red lines correspond to a putative normal distribution with same mean and variance.

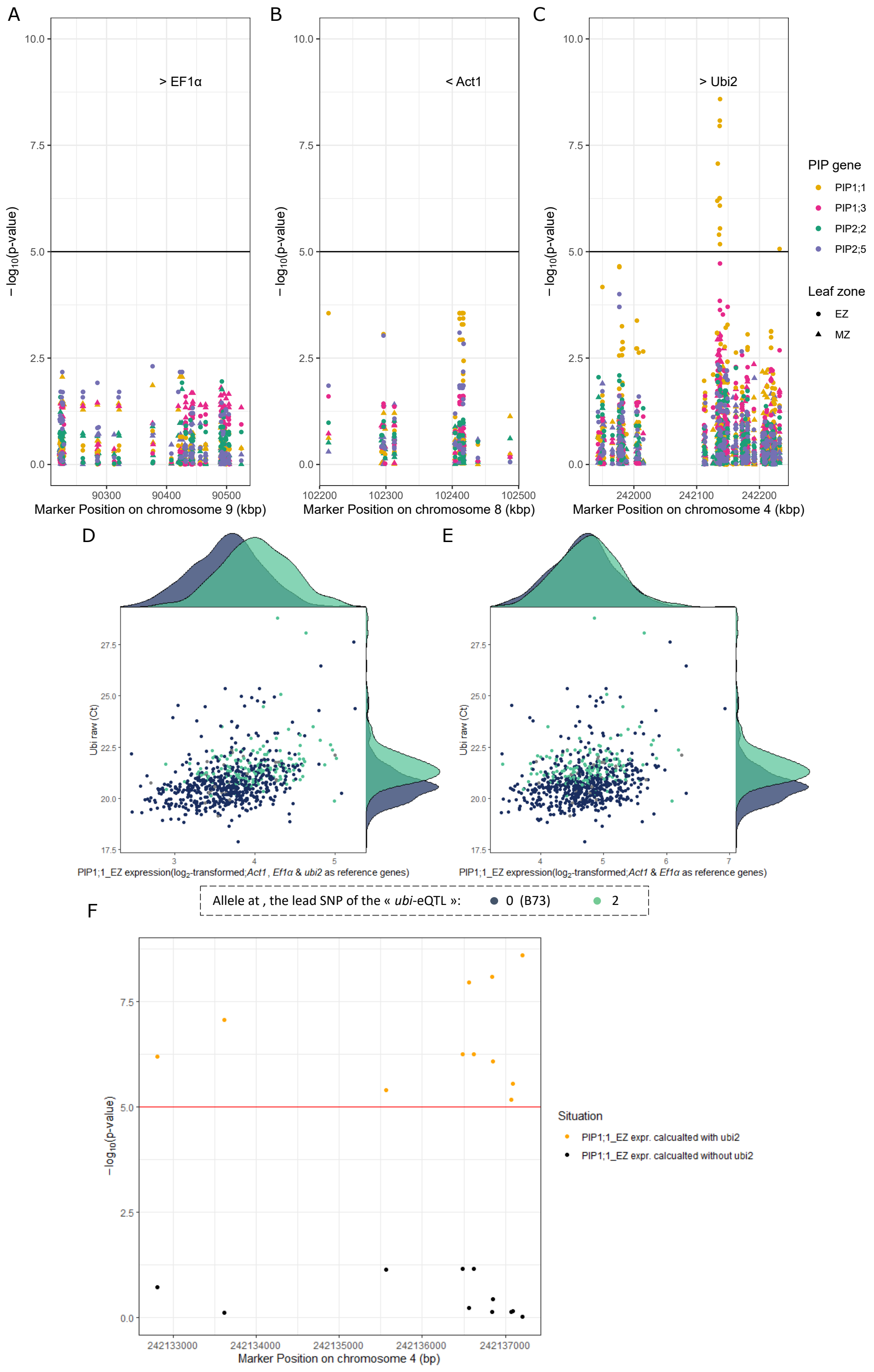

Supplementary Figure S3: Endogenous control gene location checking. Manhattan plots for all datasets centered on RT-qPCR reference gene loci. Graphs show 30 kb regions centered on the three genes used as endogenous control in the RT-qPCR: A, elongation factor 1 α, B, actin 1, C, ubiquitin 2. The genes are labeled with short names in black (black lines at 9). The arrows indicate the 5’-3’ gene orientation. The different datasets are differentiated by a combination of colors (PIP genes) and shapes (leaf zones). The horizontal black line indicates the significance threshold. D and E, show the PIP1;1 gene expression in the EZ before and after the removal of ubi as a reference gene, respectively, plotted against the ubi raw RT-qPCR data (Ct), colored according to the allele at AX-91641079, the lead SNP of the ubi-eQTL. Densities for both subpopulations defined by the allele at AX91641079 are shown along the plot edges. F is a manhattan plot showing the -log10(p-value) and the locations of markers located in the ubi-eQTL before (in orange) and after (in black) the removal of ubi as a reference gene.

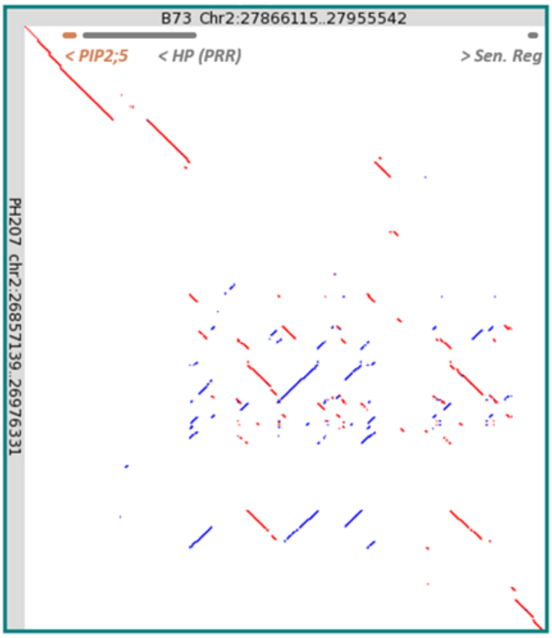

Supplementary Figure S4: B73-PH207 genome collinearity upstream of *PIP2;5*. Alignment of the B73 and PH207 genome sequence for the ~ 100 kb (sequence length selected so that collinearity was recovered at the end again) upstream of *PIP2;5*. Gene positions were indicated along the horizontal (B73) axis: *PIP2;5* is colored in orange (top left), while the two upstream genes are colored in gray. The collinearity was lost soon after the first gene upstream of *PIP2;5*. Notably, the ~6 kb indel within *hypothetical protein (HP) (PPR)* gene is visible as the horizontal shift between the two large conserved regions on the top left. Alignment and graph were obtained from LAST (http://lastweb.cbrc.jp/).

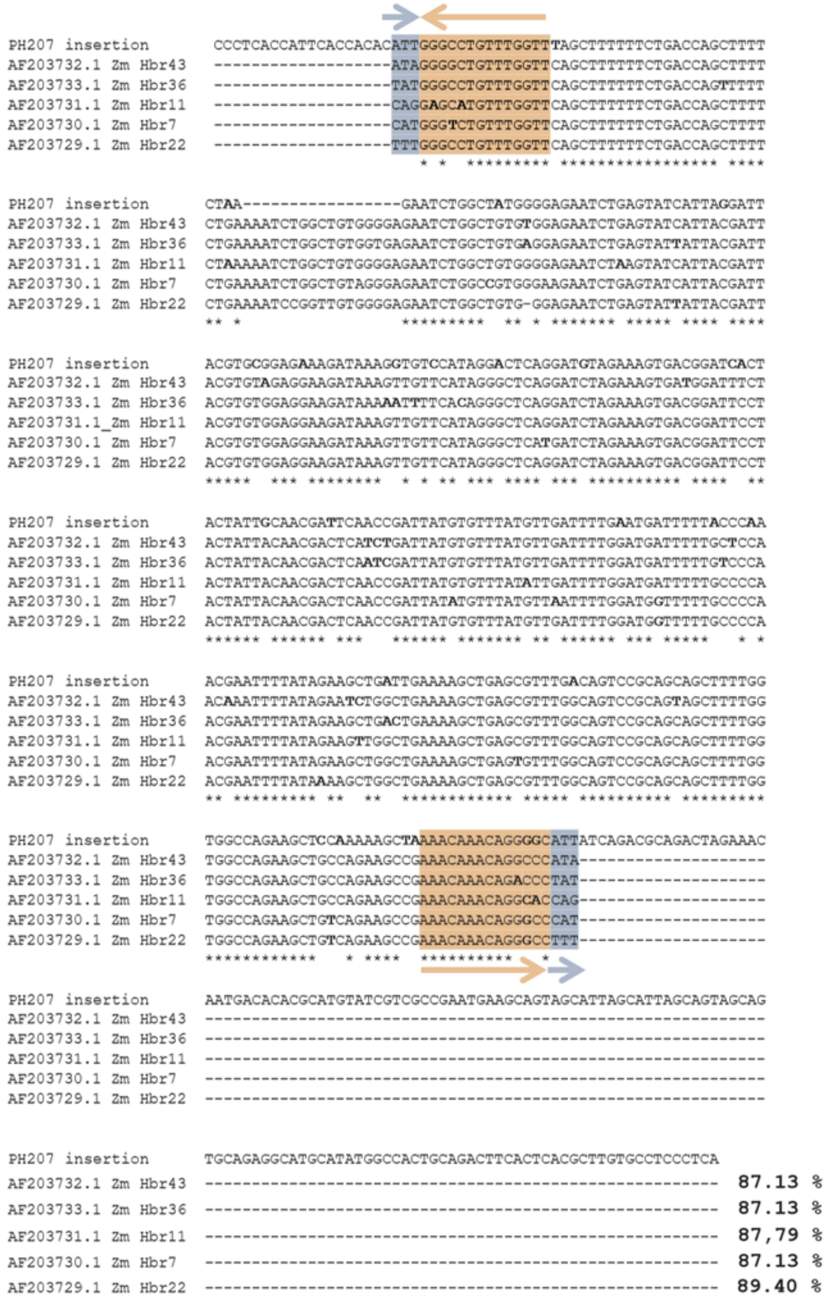

Supplementary Figure S5: The indel present in PH207 ~430 bp upstream of the *PIP2;5* start codon contains a MITE. Sequence alignment of the PH207 indel sequence (shown in the 5’-3’ sense relatively to the gene orientation) and several other *Heartbreaker* MITEs found among the results of a classical blast (NCBI) and described in Zhang *et al.* (2000). Long orange arrows indicate the characteristic terminal inverted repeats and the short blue arrows indicate the target site duplications. Respective percentages of sequence identity between the PH207 indel sequence and the aligned *Hbr* are shown at the end of the alignment. The * indicates positions where the aligned sequences are identical.

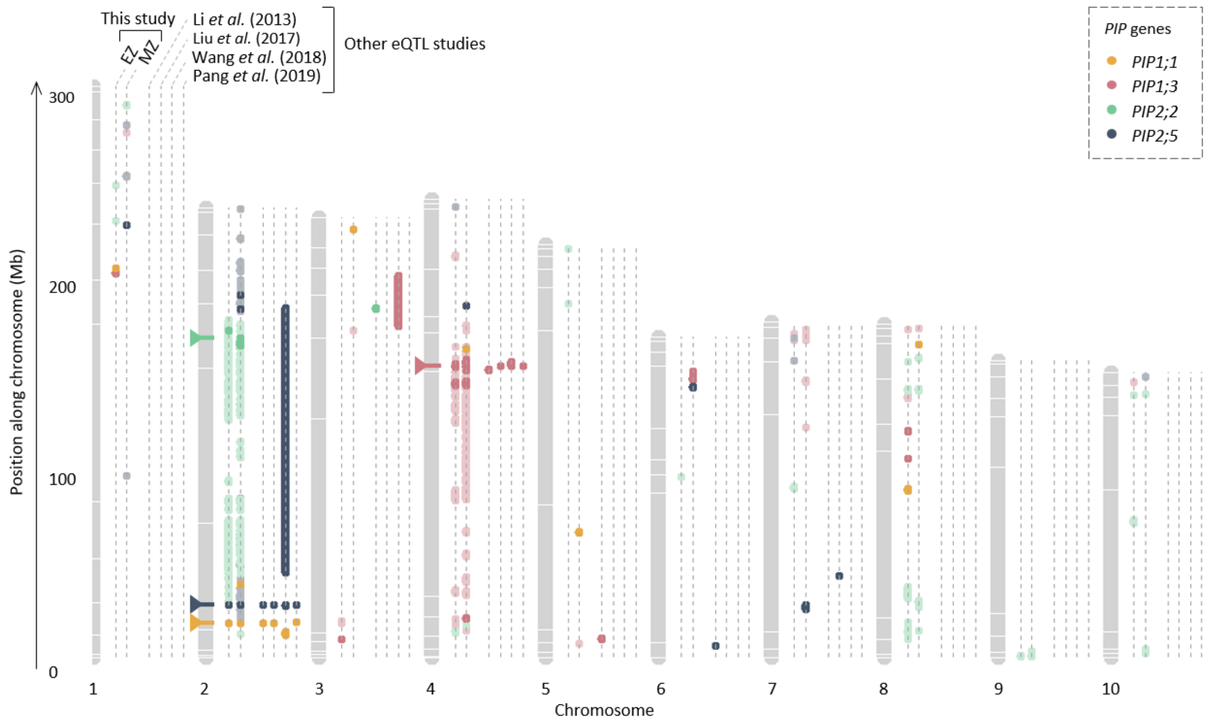

Supplementary Figure S6: *PIP* genes eQTLs. Compilation of eQTLs gathered in this work and other published eQTL studies (Li *et al.*, 2013; Liu *et al.*, 2017a; Wang *et al.*, 2018; Pang *et al.*, 2019), mapped along the genome. Chromosomes are drawn to scale in gray, with white horizontal lines indicating bin subdivisions and dark gray areas figuring centromeres. Colored arrow heads and lines indicate the position of the four *PIP* genes. The eQTLs detected are mapped on parallel dashed gray lines shifted to the right of each chromosome according to their physical boundaries. Each line represents either a leaf zone (EZ and MZ) regarding the data from this work or each of the other studies. The eQTLs (defined by the LD-window method) are colored according to the *PIP* gene concerned. Each continuous line represents a unique eQTL, whose physical range may sometimes be very large. Overlapping lines (barely visible, shown by irregular borders) indicate the presence of adjacent eQTLs. Regarding the data from this work, for cases where covariate GWAS were implemented, eQTLs coming from the initial GWAS (except for the local one) were colored in lighter shades. In view of the pyramiding eQTL and tail patterns, suggesting the need for subsequent covariate steps for both *PIP2;2* datasets, only the most significant eQTLs and the local eQTL detected for *PIP2;2*_EZ are normally colored, while the others are colored in a lighter shade to indicate the uncertainty about them. Physical positions of the eQTLs are based on the B73 reference genome RefGen_v2. Physical positions from Pang *et al.* (2019) and Li *et al.* (2013) were translated from RefGen_v3 to RefGen_v2 with the appropriate assembly converter from EnsemblPlant (http://ensembl.gramene.org/Tools/AssemblyConverter). Physical positions from Wang *et al.* (2018) had to be inferred from genetic positions.

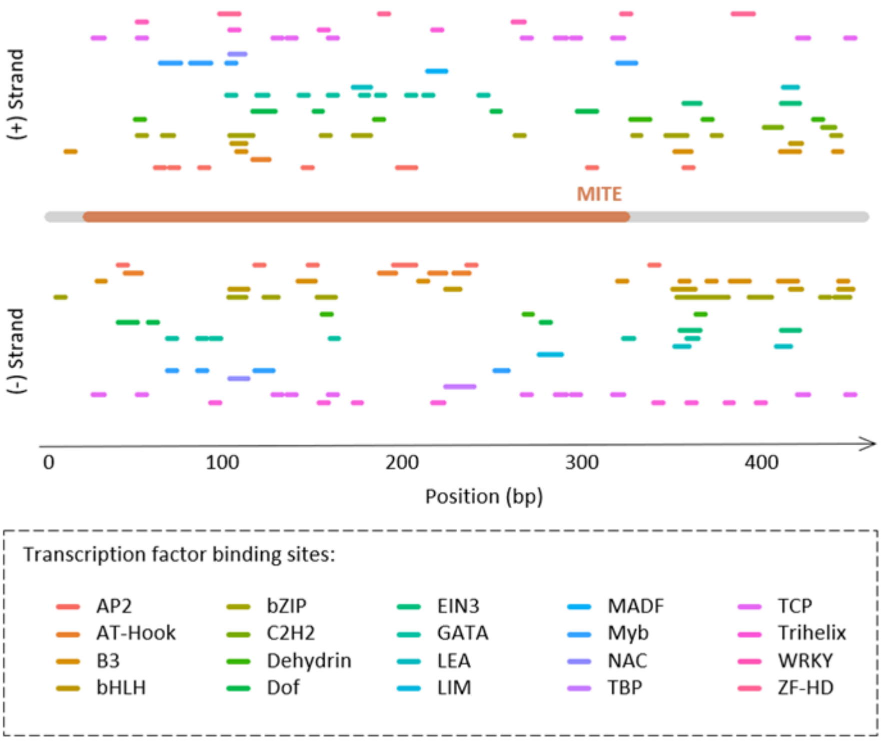

Supplementary Figure S7: Predicted transcription factor binding sites within the MITE-containing indel sequence. Many potential TFBS motifs were found within the MITE-containing indel sequence, and within the MITE itself as well (indicated as an orange line). A non-exhaustive list of TFBS types is shown. They are colored according to their binding TF type and mapped along the schematic indel (shown in the 5'-3' orientation relative to the *PIP2;5* gene). They are located above or below the schematic indel depending on their location on the plus or minus strand. They are slightly offset along the vertical axis for the sake of clarity. TFBS prediction was performed with PlantPan 2.0 (Chow *et al.,* 2016), using the databases available for all plant species altogether.

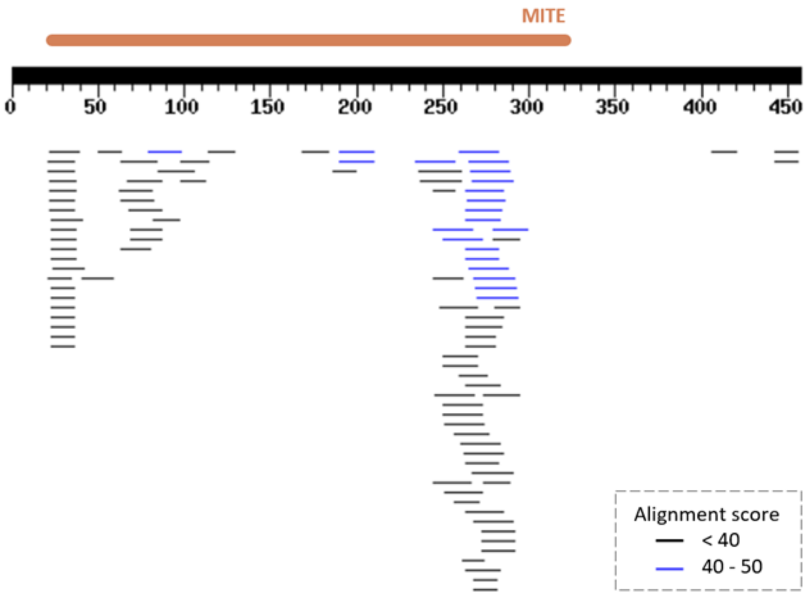

Supplementary Figure S8: small RNA aligning on the MITE-containing indel sequence. Many small RNA sequences were found to align on the indel sequence, and especially within the MITE (shown in orange). The indel is shown in the 5'-3' orientation relative to the *PIP2;5* gene. Checking of the individual alignment showed some perfect matches. Some of them included polymorphisms specific to the MITE of interest. Adapted from an original figure and data from http://sundarlab.ucdavis.edu/smrnas/.
