# Supplementary material for: Genetic variability of aquaporin expression in maize: From eQTLs to a MITE insertion regulating PIP2;5 expression
Source: Plant Physiol. 2024 Jun 5;196(1):368–84. doi: 10.1093/plphys/kiae326 (PMC11376376; doi:10.1093/plphys/kiae326)
Supplement: kiae326_Supplementary_Data [file kiae326_supplementary_data.zip › Supplemental Appendix S1.pdf]

Supplementary Appendix S1: Analysis of the putative polymorphisms within the primer sequences.

***PIP* gene primer sequences**

We investigated the presence of polymorphisms among the sequences targeted by the qPCR primers and evaluated the impact of such pinned polymorphisms on the mapped local eQTLs. We exploited the genotyped SNPs and the public availability of sequenced genomes corresponding to eight lines of the DROPS panel. They were accessed from MaizeGDB and concerned B73 (V1 : Schnable et al. (2009) ; V4 :Jiao et al. (2017), PH207 (Hirsch *et al.*, 2016), Mo17 (Sun *et al.*, 2018), B104, B97, MS71, NC358 and Oh43 (Hufford *et al.*, 2021). Several polymorphisms were detected within the primer regions. Some of them were present in the SNP marker collection and had consequently been genotyped or imputed for the whole panel. Association with, and putative influence on, the respective detected local eQTLs were then assessed. Others were only noticeable from the comparison of available sequenced genomes. Therefore, in order to reduce uncertainties in these cases, 20 lines were selected for targeted sequencing. Choice was directed, as much as possible, by divergence in the allele at the local eQTLs of interest and diversity in population structure groups.

Three genotyped SNPs were identified in the primer pair targeting *PIP1;1*. Individually, none of them seems to have an impact on the local eQTL detected, and they were, indeed, not even detected as significant in the GWAS (data not shown). Four combinations (out of eight possible) were found among the panel, distributed among all population structure groups (Fig. A1), but no striking correlations were observed with the local eQTL alleles (data not shown).

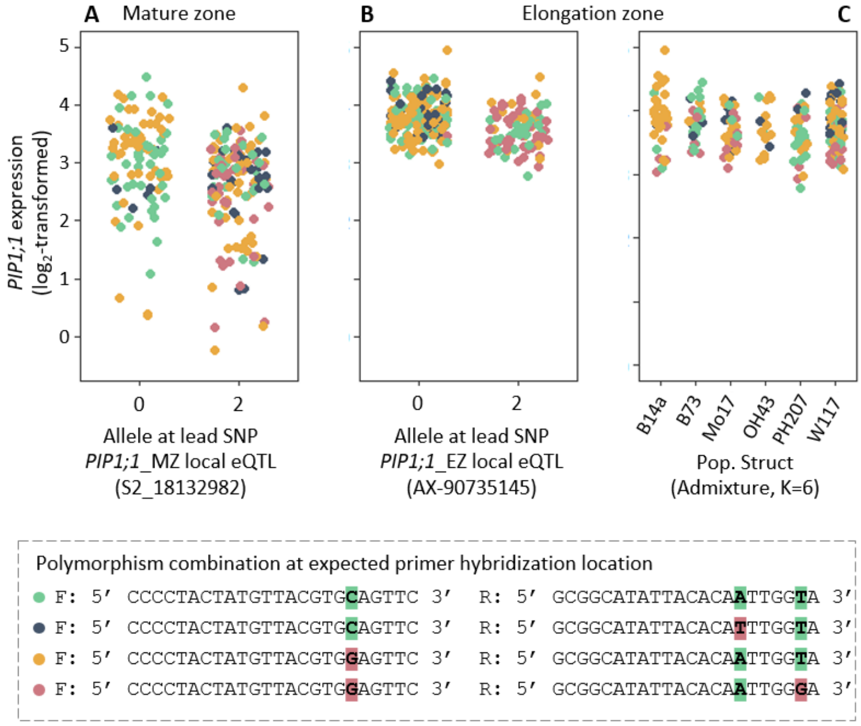

Figure A1: *PIP1;1* primer polymorphisms and local eQTLs. *PIP1;1* expression (log_2-transformed) in the mature (**A**) and elongation zone (**B**), according to the alleles at the cognate local eQTL lead SNP, or according to the admix groups (**C**, EZ data). Lines are colored according to the combination of primer polymorphisms they harbor. Detected polymorphisms are highlighted in green and red, depending on their allele. Sequences are presented linearly in the 5'-3' orientation relatively to the gene orientation. For sake of clarity, three lines with heterozygous genotypes for at least one SNP were removed. Presented primer sequences are hypothetical: known polymorphisms come from the SNP collection used for the GWAS, and the comparison of eight available genome sequences. Other undetected polymorphisms may exist. B73 and the primers that were used match the first, green, combination. F and R stand for forward and reverse, respectively.

Regarding *PIP1;3*, one genotyped SNP (AX-90895950) was mapped within the forward primer region. In addition, an indel of two A bases was spotted at the location of the reverse primer. Among the reference genomes and the additional 20 sequenced lines, three possible combinations of the polymorphisms were detected (Fig. A2). The genotyped SNP within the forward primer region was perfectly associated with the local eQTL obtained for the EZ, as it was the lead SNP of this particular eQTL. Interestingly, it was not as much associated with the local eQTL detected for the MZ datasets (Fig. A2, B). Also, the occurrence of the two alternative alleles seems fairly balanced among the population, irrespectively from the population structure (Admixture, K=6) (Fig. A2, D). The presence of a different MZ local eQTL,

as well as the detection, in both cases, of secondary local eQTLs when setting the first ones as covariates, suggested the presence of multiple causal polymorphisms combined in different haplotypes, hence supporting the hypothesis that the SNP is indeed forcing an unreal variation in expression but other causes of variation still exist. Among the additional selected sequenced lines, the second, yellow, combination (Fig. A2, A) was poorly represented. However, in each case, the second local eQTL was more driven by the subpopulation defined by the non-B73 allele at the first local eQTL and, consequently, by the subpopulation presumably associated

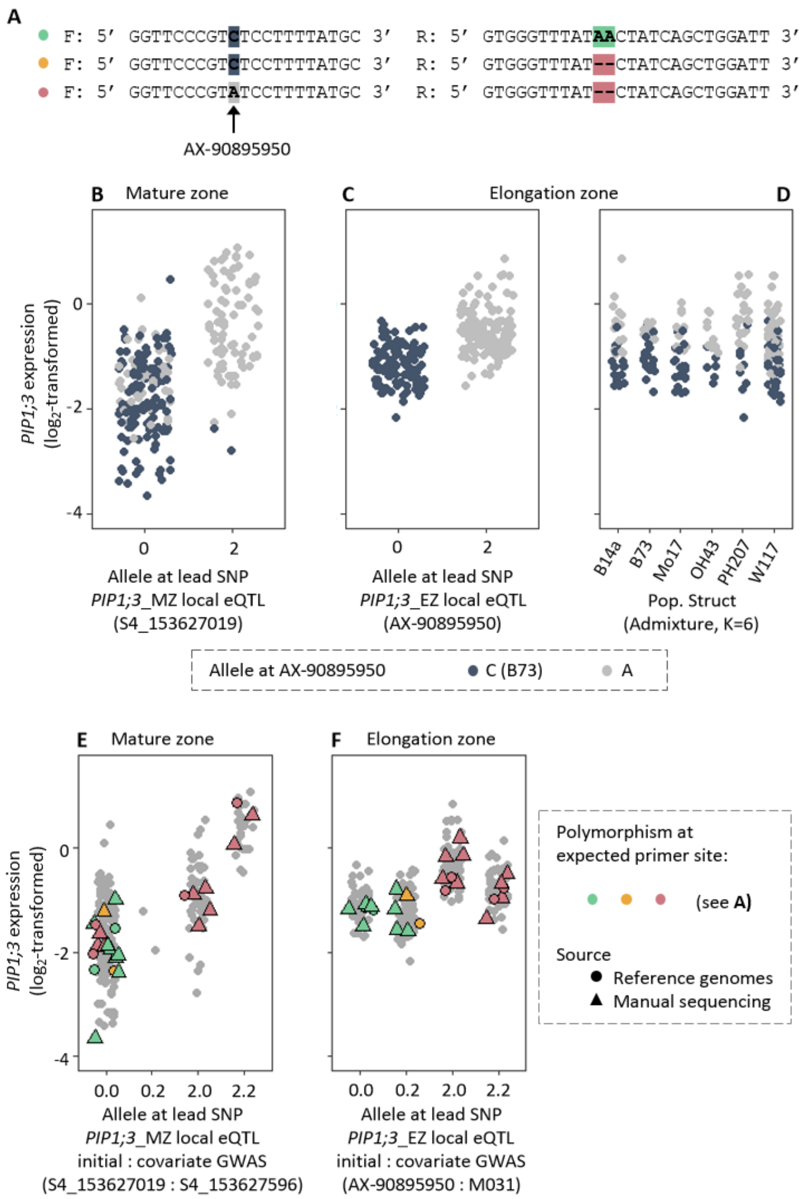

to the non-B73 allele at the forward primer SNP (AX-90895950; red combination) (Fig. A2, E and F), suggesting that the AA indel is not involved in those secondary local eQTLs.

Figure A2: *PIP1;3* primer polymorphisms and local eQTLs. Three different combinations of primer polymorphisms found among the sequenced maize lines, coming from available genome sequences matching the panel, as well as manually sequenced lines (from hybrid cDNA samples) **(A)**. The AX90895950 SNP on the forward primer is highlighted in blue (B73 allele) and or gray (alternate allele). Other detected polymorphisms are highlighted in green and red, depending on their allele. The green allele corresponds to B73. A color code (green, yellow, red) is associated to each combination. Sequences for forward (F) and reverse (R) primers are presented linearly in the 5'-3' orientation relatively to the gene orientation. UH007 and the primers used for the RT-qPCR correspond to the red combination, while B73 corresponds to the green one. *PIP1;3* expression (log2-transformed) in the mature **(B)** and elongation zone **(C)**, according to the allele at the cognate local eQTL lead SNP, or according to the admix groups **(D)** (EZ data). Lines are colored according to the allele at AX-90895950. *PIP1;3* expression (log2-transformed) in the mature **(E)** and elongation zone **(F)**, according to the allelic series defined by the combination of alleles at the two cognate local eQTL, detected by initial and covariate GWAS. The first position is always the initial local eQTL lead SNP, while the second is the covariate local eQTL lead SNP. Lines whose sequences are known at the expected primer annealing locations are emphasized. Symbol shapes figure the sequence origin, while colors indicate the combination of primer polymorphisms they harbor. B73 matches the first, green, combination while primers used and UH007 matches the third, red, combination.

No genotyped marker was located within the *PIP2;2* primer region. Nonetheless, reference genome and additional sequencing allowed the detection of several polymorphisms (Fig. A3). Regarding the reverse primer location, two more alternative alleles were identified in place of the used primer sequence. These different alleles, located within a large C-repeat and at the external 5' end of the primer, are thought to likely be negligible. On the contrary, genome and targeted sequences alignment revealed a very different sequence at the expected target site of the forward primer. Data from the available genomes and manually sequenced lines indicated that the local eQTL detected (identical lead SNP for MZ and EZ datasets) was strongly associated with the repartition of the alleles at this forward primer location. Also, it makes sense that the very divergent allele at the forward primer location was associated with the unfavorable eQTL allele, the lower - but still real - expression being presumably attributed mainly to the UH007 allele (harboring the correct, green, combination in Fig. 25). Fortunately, if we consider the local eQTL to perfectly reflect this forward primer location allele divergency, and because the local eQTL (lead SNP PZE-102122092) unfavorable allele concerned only 29 lines, the remaining population is still considerable. Therefore, we discarded the 29 lines defined by the unfavorable PZE-102122092 allele from the *PIP2;2* analysis and only the 220 remaining ones, harboring the favorable allele, were used for the GWAS (three lines genotypes as heterozygous at this location were discarded) (Results, Fig. 6B). Only the GWAS results obtained from this subpopulation will be considered for *PIP2;2* (referred to as subpopulation/Subpop GWAS). This makes sense in view of the unbalanced

proportion of both subpopulations (favorable vs. unfavorable alleles at PZE-102122092) within the panel, so that this larger favorable subpopulation drove most of the results.

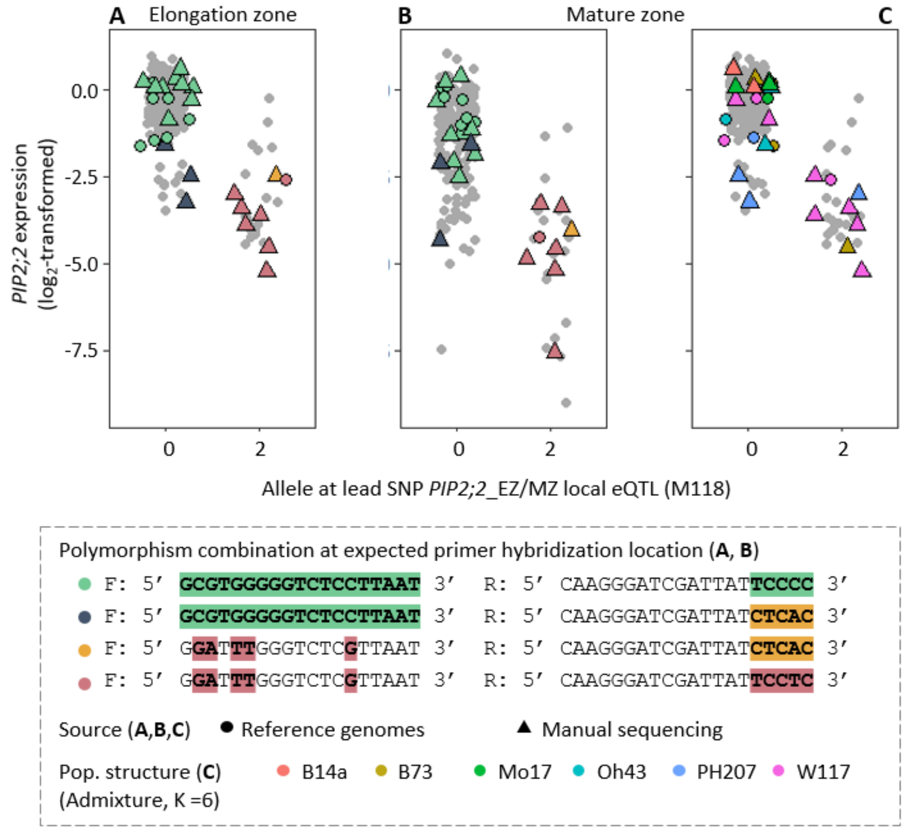

Figure A3: PIP2;2 primer polymorphisms and local eQTLs. *PIP2;2* expression (log2-transformed) in the mature **(A)** and elongation zone **(B & C)**, according to the allele at the cognate local eQTL lead SNP (M118 in v2 and PZE-102122092 in v4, same for MZ and EZ datasets). Lines whose sequences are known at the expected primer annealing locations are emphasized. Symbol shapes figure the sequence origin (either published genome sequence or manual sequencing, from hybrid cDNA samples - 19 sequenced samples out of the 20 selected ones), while colors indicate the combination of primer polymorphisms they harbor **(A & B)**, or according to the admix population structure groups **(C)**. For sake of clarity, lines with heterozygous genotypes at the local SNPs were removed (three in each case). Sequences for forward (F) and reverse (R) primers are presented linearly in the 5’-3’ orientation relatively to the gene orientation. Both B73 and the primer that were used match the first, green, combination. UH007 matches the second, blue, combination.

No polymorphism was identified among the regions targeted by the RT-qPCR primers regarding the *PIP2;5* genes, either in the marker collection or when comparing genomes.

It is noteworthy that a few polymorphisms were identified within the reference gene primers. One point mutation was identified within the *Ubi* forward primer, at 5 bases before

the 5’ end of the primer. This SNP was genotyped by GBS (S4_236883474). Its low MAF (0.131) and its non-significant -log10(p-value) (0.054, while the cutoff value was set at 5) in the initial GWAS for *PIP1;1*_EZ, suggests it was not responsible for, or associated with, the problematic *ubi*-eQTL presented above. A point mutation was identified in the middle of the reverse primers targeting *Ef1α,* when comparing available genomic sequences. A punctual mutation was also identified at the 5’ end of the *Act1* forward primer. None of them were genotyped. No other SNP within the primer regions was identified among the collection.

Materials and methods

Following the observation of polymorphisms located within the initial RT-qPCR primer sequences among reference genomes, cDNA from 20 line x UH007 hybrids (where line = A374, A375, B100, B108, B110, B14a, B89, EP29, F04402, F7058, F712, LH59, Lo1063, Lo1095, Lo1124, Oh50B, PHJ40,PHK29, UH_2500, UH_P064) were selected to verify the sequence at the location of the RT-qPCR primers used for PIP1;3 and PIP2;2 expression quantification. Primers used to amplify the surrounding region are listed in Table A1. PCR products amplified with Q5 polymerase (New England Biolabs) were sequenced (Microsynth). When necessary, the distinction between double trace signals (arising from the line of interest and UH007) was performed manually or by using Poly Peak Parser (Hill *et al.*, 2014). UH007 allele was identified by cloning the PCR product from a B73xUH007 cDNA in pGem-T_EASY (Promega) and sequencing several clones.

Table 7: Primers used for RT-qPCR primer sequence verification (named PIPXX_Checkseq_FX). Primers are shown in their 5’-3’ orientation.

Gene	Forward primer	Reverse primer
PIP1;3	F: TGAGACATGTGGGGAATGTA<br>F2: TCAACCCGGCTAGGAGC	R1: ATCGATTCCACTTGGATGAAA
PIP2;2	F: GCGCGAGGGAGGAGAATAAG	R: GAGGCAAGAGGTTTCTCACTG
	F2: GTCGCTACATAATAACG	R2: GCAACGCGTGATGAGCAGA

References

Hill, J.T., Demarest, B.L., Bisgrove, B.W., Su, Y., Smith, M. and Yost, H.J. (2014) Poly peak parser: Method and software for identification of unknown indels using sanger sequencing of polymerase chain reaction products. *Developmental Dynamics*, **243**, 1632–1636.

Hirsch, C.N., Hirsch, C.D., Brohammer, A.B., et al. (2016) Draft Assembly of Elite Inbred Line PH207 Provides Insights into Genomic and Transcriptome Diversity in Maize. *Plant Cell*, **28**, 2700–2714.

Hufford, M.B., Seetharam, A.S., Woodhouse, M.R., et al. (2021) De novo assembly, annotation, and comparative analysis of 26 diverse maize genomes. *Science (1979)*, **373**, 655–662.

Jiao, Y., Peluso, P., Shi, J., et al. (2017) Improved maize reference genome with single-molecule technologies. *Nature*, **546**, 524–527.

Schnable, P.S., Ware, D., Fulton, R.S., et al. (2009) The B73 Maize Genome: Complexity, Diversity, and Dynamics. *Science (1979)*, **326**, 1112–1115.

Sun, S., Zhou, Y., Chen, J., et al. (2018) Extensive intraspecific gene order and gene structural variations between Mo17 and other maize genomes. *Nat Genet*, **50**, 1289–1295.
