# Supplementary material for: Genetic variability of aquaporin expression in maize: From eQTLs to a MITE insertion regulating PIP2;5 expression
Source: Plant Physiol. 2024 Jun 5;196(1):368–84. doi: 10.1093/plphys/kiae326 (PMC11376376; doi:10.1093/plphys/kiae326)
Supplement: kiae326_Supplementary_Data [file pp.00279.2024-s05.pdf › pp.00279.2024-s05.pdf]

Plant Physiology®

# Genetic variability of aquaporin expression in maize: from eQTLs to a MITE insertion regulating PIP2;5 expression

Laurie Maistriaux, Maxime Laurent, Linda Jeanguenin, Santiago Alvarez Prado, Joseph Nader, Claude Welcker, Alain Charcosset, Francois Tardieu, Stéphane Nicolas, and Francois Chaumont

**Review Timeline:**

| | |
|---|---|
| Submission Date: | 18-Sep-2023 |
| Editorial Decision: | 02-Nov-2023 |
| Resubmission Received: | 28-Feb-2024 |
| Accepted: | 02-May-2024 |

02-Nov-2023

Prof. Dr. Francois Chaumont
UCLouvain
Louvain Institute of Biomolecular Science and Technology
Louvain-la-Neuve B-1348
Belgium

RE: Genetic variability of aquaporin expression in maize: from eQTLs to a MITE insertion regulating PIP2;5 expression

Dear Dr. Francois Chaumont:

Thank you for giving us the opportunity to review your manuscript for publication in the Plant Physiology. The reviewers have found the manuscript of interest, but have raised a number of concerns that will require your attention. As these concerns are of some gravity and will entail substantial changes to the manuscript, you are likely to need additional time to address them. I must therefore reject the manuscript in its present form. Nonetheless, if you are able to address the points raised in review, I would welcome your resubmission of a manuscript that incorporates your responses.

In particular, I direct your attention to the following comments for consideration in a resubmitted manuscript.

Points 1-3 from reviewer #2, point 2 from reviewer #1 together with the results you present on potential snp effects on target/reference genes and dealing with LD effects lead to concerns regarding the robustness and broader significance of the method used. These aspects together with the other points raised should be addressed and discussed more fully in a revised version.

An alternative option I would support, but not require, could to reduce the emphasis on the GWAS by further integration of the findings with other physiological data collected on the panel that are linked to the findings (as you discuss). This would need to go beyond those you have previously published on this gene (OX / KO) linking to growth and water relations but I expect with the rich data collected there may be various options.

Please note that any resubmission will be treated as a new entry to the system and is likely to go out for review.

If you do have any queries or concerns, you are welcome to contact me in the first instance.

Thank you again for allowing Plant Physiology to review your work. We look forward to hearing from you soon.

Sincerely,

Editor, Plant Physiology

---------------------------------------------------------------------------- Reviewer comments:
Reviewer #1 (Comments for the Author):

The ms by Maistriaux et al. describes the analysis of the genetic control of aquaporins (PIP) gene expression based on a diversity panel in maize. The approach is based on qRT-PCR of four aquaporin genes on two leaf tissues. These quantitative traits are then analyzed by GWAS using pre-existing genotypic data. A major expression QTL is then characterized in more detail and a likely causative polymorphism (transposon insertion) is identified, although quite preliminarily.
The work is well organized around a very clear subject and objective, ie. shed light on the variability of PIPs expression and their genetic control in terms of eQTL based on GWAS. The methods chosen, including statistics, are appropriate and at the state of the art. Results are discussed well.
Altogether, the work is interesting and provides a step forward in understanding how complex is the genetic control of gene expression for this important gene family.
I have a few comments, addressing a few weak points.
General comments

1. The authors distinguish between, and mostly discuss about, local or distant eQTLs, however in literature is more common to read about cis or trans eQTLs. I guess a couple of sentences providing some explanations about this would be welcomed in DISCUSSION.
2. The authors do not really address the point that, because of the nature of plant materials (F1 hybrids), the quantified RNA for each gene is actually a mixture of two populations of RNA molecules, one from the tested genotype and one from the tester. Could you please comment on this?

3. I would give more attention to Table heads and Figure Captions, inlcuding their titles. Quite generally, Table heads seem long to me and some text can be moved as Table footnotes. One Table title was too short, see below.

Specific comments
P6, line 147. Coincided
P6 Line 152. 'in most cases they were far less significant'. It think this is too general and not scientific. I guess it should be possible to test this statistically.
P7, Line 199. Gene names in italics
Figure 7B, upper part (the one showing the PIP and PPR genomic region) is probably useless, and it seems a repetition of what shown in figure 6E.
Caption of Figure 7. The last sentence about the agarose gel runs can be deleted as it seems unnecessary to me.
P11 L310. blur or blurred?
P11 L325-326. I do not think the expectation is that there is no variation in PIP expression agronomic lines. Please consider to avoid this comment.
Title head (in bold) of Table 2 is too short (eQTLs)! I suggest to extend it as a full sentence. On the contrary, most of the information provided in the current head should go as table foot note.
Other portions of Table 2 require attention: for instance 'Nb' is not the correct short version for 'Number', it should be No. (if I am not wrong).

Reviewer #2 (Comments for the Author):

In this article, the authors analyze expression patterns of four plasma membrane-intrinsic proteins (PIPs) across a diversity of European-adapted maize germplasm to identify genomic regions regulating the expression of these genes (eQTLs). Principal component analysis based on heterotic subgroupings revealed differential expression patterns among the selected PIPs, with a distinct separation between B73/Oh43 and PH207-derived germplasm. Subsequent GWAS using several modeling approaches identified a total of 588 eQTLs in proximal and distal locations relative to the genes, with proximal eQTLs contributing the largest, most significant effect. Of the eQTLs identified, a Miniature Inverted-repeat Transposable Element (MITE) insertion in the promoter region of PIP2;5 was present in PH207, but absent in B73 or Oh43, and was selected as a target for additional characterization. Constructs containing the native B73 and PH207 versions of the PIP2;5 promoter driving mVenus, in addition to edited versions of the promoters that inserted or removed the putatively causal MITE, were used to transiently transform maize leaves; confocal microscopy showed a significant reduction in fluorescence intensity whenever the MITE was present, supporting the claim that this mutation underlies the reduced expression of this eQTL. While the findings were exciting, some aspects of the methodology were confusing/questionable and did not produce compelling results.

Major Points:
1. Lines 65-69 are copied almost verbatim from the Hachez et al. (2008) source cited parenthetically in Line 65. Appropriate paraphrasing should be used when referencing source material to avoid plagiarism, however unintentional it may be.
2. The significance levels used for GWAS are inappropriate, with an initial significance threshold arbitrarily set to 5 and then set to 12 for covariate GWAS (Line 564-565; 171-172). If an arbitrary cutoff for significance is chosen, it has generally been considered as p < 5 x 10-8. However, a more suitable approach for establishing significance would be to conduct a Bonferroni correction based on the number of markers used for the association study. In addition, because different significance thresholds are used throughout the manuscript, please make sure to indicate significance thresholds for each test (such as in Figure 8).
3. The authors are using RefGen_v2 of the maize genome, which is more than a decade old and several versions outdated. The SNP locations should be migrated to a new version for the publication. No doubt this will clean up analyses, as the authors point out in line 163-166 that LD between distant locations could be caused by assembly error.
4. How were the 14 lines selected for analysis and inclusion in Table 4? Evaluating the PIP2;5 promoter strength with and without the MITE insertion through use of fluorescence imaging provides evidence (although noisy) to support that this mutation underlies the eQTL. However, Table 4 shows a seemingly clear delineation in PIP2;5 expression levels in the step change between Mo17 and B100. A374 and B97 are below this step change, yet they also have the MITE insertion. Indeed, A374 appears to share the same haplotype of PH207 for all potential polymorphisms. The question is, does the expression across the larger panel correlate with the presence/absence of the MITE?

Minor Points:
1. The authors report that a total of 759,659 markers were selected with a MAF > 0.05 (Line 137, 551-553); however, it is not stated whether all these markers were informative. Are they? Additional information for SNP selection would be useful to understand how they were selected for the analysis.
2. In the Discussion section the authors need to address the large number of eQTLs identified compared to other studies. Does this relate to false positives or marker selection? This would add to the rationale given in Lines 304-321 is insufficient.
3. Given the ubi-eQTL identified, it seems that the most straight-forward test would be to drop ubi2 as a control gene and rerun the analysis. If the eQTL disappears it is likely an artifacte and the Ubi gene is not a good "control gene". However, if it remains, the authors should reconsider dismissing the eQTL hit on ubiquitin2 in relation to PIP1;1_EZ (Line 188-190. The authors should

at least note previous literature linking PIP cycling to the 26S proteasome pathway (see Lee et al., 2009; https://doi.org/10.1105/tpc.108.061994). As PIP1;1 expression is much higher than all other maize aquaporin genes, it is not unreasonable to consider that cycles in protein levels and protein degradation patterns may be differentially regulated than other PIPs, thereby showing a signature at ubi2 even when no other aquaporins do.

4. Line 272-273: Reference is made to an absent Figure 9; should this be Figure 7B?
5. Line 499: 252 hybrids are reported as included in this panel. Throughout the manuscript reference is made to admixture analysis supporting 6 heterotic groups. List in methods how many lines fell into each of these heterotic groups and whether any groups were over/underrepresented.
6. Line 512: B104 is an inbred and is otherwise correctly denoted throughout the rest of the manuscript.
7. Line 633: Potential typo in reporting microscope model used (Zeiss LSM70).
8. Provide GeneIDs for the different genes discussed (aquaporins, ubi2, etc.), using either B73 RefGen_v3 or Zm-B73-REFERENCE-NAM-5.0 annotations as the highest quality GeneIDs.

## Responses to the editor and reviewers

*Please see our responses in italic.*

**Comment from the Monitoring editor:**

Points 1-3 from reviewer #2, point 2 from reviewer #1 together with the results you present on potential snp effects on target/reference genes and dealing with LD effects lead to concerns regarding the robustness and broader significance of the method used. These aspects together with the other points raised should be addressed and discussed more fully in a revised version.

*Response: As requested, we addressed the different concerns raised by the reviewers as detailed below. We notably convert the SNP positions to version 4 and reanalyzed all our data to provide a more up-to-date version of our eQTL positions. We also addressed the potential SNP effect on target/reference genes (see the response to the point 2 from reviewer #1).*

An alternative option I would support, but not require, could to reduce the emphasis on the GWAS by further integration of the findings with other physiological data collected on the panel that are linked to the findings (as you discuss). This would need to go beyond those you have previously published on this gene (OX / KO) linking to growth and water relations but I expect with the rich data collected there may be various options.

*Response: This suggestion to integrate more physiological data was indeed highly relevant. However, this would require extensive analysis and the acquisition of new experimental data. We are currently part of a collaborative project in which such approaches are considered.*

**Reviewer #1 (Comments for the Author):**

The ms by Maistriaux et al. describes the analysis of the genetic control of aquaporins (PIP) gene expression based on a diversity panel in maize. The approach is based on qRT-PCR of four aquaporin genes on two leaf tissues. These quantitative traits are then analyzed by GWAS using pre-existing genotypic data. A major expression QTL is then characterized in more detail and a likely causative polymorphism (transposon insertion) is identified, although quite preliminarily.

The work is well organized around a very clear subject and objective, ie. shed light on the variability of PIPs expression and their genetic control in terms of eQTL based on GWAS. The methods chosen, including statistics, are appropriate and at the state of the art. Results are discussed well.

Altogether, the work is interesting and provides a step forward in understanding how complex is the genetic control of gene expression for this important gene family.

I have a few comments, addressing a few weak points.

General comments

1. The authors distinguish between, and mostly discuss about, local or distant eQTLs, however in literature is more common to read about cis or trans eQTLs. I guess a couple of sentences providing some explanations about this would be welcomed in DISCUSSION.

*Response: We now justified the use "local" and "distant" terms in the results (lines 152-155). "Local and distant terms were preferred to cis and trans terms, which infer a biological mode of action. By using local and distant terms, we avoid making assumptions about their role (Blein-Nicolas et al., 2020). "*

2. The authors do not really address the point that, because of the nature of plant materials (F1 hybrids), the quantified RNA for each gene is actually a mixture of two populations of RNA molecules, one from the tested genotype and one from the tester. Could you please comment on this?

*Response: The 252 inbred lines of the panel were all crossed with a same unique line (UH007) to obtain the 252 F1 hybrids of the hybrid panel. As all hybrids share a common parent, the phenotype (here, the RNA abundance) of a given hybrid can be associated to its specific parent, i.e. a given line within the panel. Indeed, one allele is constant between all hybrids (the one coming from the tester), the only allele that varies between hybrids is the one coming from the lines. Either the lines share the same allele as the tester (homozygous), or the lines do not share the same allele (heterozygous). In the absence of allele specific expression (ASE), we expect the abundance of RNA molecules of the tester to equal the RNA abundance of the line and, therefore, no variation in RNA molecule abundance between hybrids homozygous for the tester allele & heterozygous (no effect). In the presence of ASE, we would expect the abundance of RNA molecule in the lines to be different from that of the tester in the heterozygotes and therefore the abundance of RNA molecule in the heterozygote to be higher or lower than that in the homozygotes (effect).*

*GWAS is then conducted accordingly considering the (homozygous) genotype of an inbred line and the phenotype of its hybrid progeny with UH007. This corresponds to a usual practice in hybrid crop genetics (eg. Rincent et al., 2014, Theor Appl Genet. 127: 2313-31 and Rincent et al., 2014 Genetics, 197: 375-87), where the common line (UH007) is referred to as "tester" to reveal the "general combining ability" of lines within a panel. It is particularly suitable in our case where UH007 belongs to the "flint" genetic group, which is complementary to the dent group of the panel, so that no hybrid is affected by inbreeding depression due to their closest genetic proximity with the tester. Since all hybrids share a same parent (UH007), it is not possible to estimate a dominance effect in our GWAS model. For each polymorphic SNP, the 252 lines of the panel either carried the same allele than UH007 (homozygous hybrid) or a different allele than UH007 (heterozygous hybrid). As a consequence, we can only test the phenotypic (mRNA abundance) difference between two genotypic contrasts. We hypothesized that there was no interaction between the tester and the inbred alleles. We mentioned it and added references in the results (lines 113-123).*

*In our study, we also tested the presence of polymorphisms among the sequences targeted by the qPCR primers (see Appendix 1) that could explain variation in RNA abundance. Annealing regions of the primers of gDNA originating from hybrids were sequenced. The sequencing results demonstrated that mutations within the primer regions for PIP1;1 did not affect the GWAS results as shown and discussed in the appendix. For PIP2;2, there was a mutation in the forward region that could have impacted the measurement in some lines. To overcome this issue, we performed a subpopulation GWAS by removing hybrids presenting the mutation in the forward primer as discussed at lines 218-224. For PIP1;3, one genotyped SNP within the forward primer was associated with the local eQTL obtained in the elongation zone, but not with the local eQTL detected in the mature zone. However, multiple other causal polymorphisms seem to exist to explain local the eQTL in the elongation zone (see Appendix 1). Finally, no polymorphism was identified among the regions targeted by the RT-qPCR primers of the PIP2;5 gene, either in the marker collection or when comparing genomes,*

*strengthening our data regarding a causal variant of the local eQTL (MITE containing indel). We transferred some information from the Appendix S1 in the main text (lines 216-218, lines 224-229).*

3. I would give more attention to Table heads and Figure Captions, including their titles. Quite generally, Table heads seem long to me and some text can be moved as Table footnotes. One Table title was too short, see below.

*Response: Thanks for the comment, we took in account this remark.*

<u>Specific comments</u>

P6, line 147. Coincided

*Corrected*

P6 Line 152. 'in most cases they were far less significant'. It think this is too general and not scientific. I guess it should be possible to test this statistically.

*Response: We did agree and removed this sentence that was not needed.*

P7, Line 199. Gene names in italics

*Corrected*

Figure 7B, upper part (the one showing the PIP and PPR genomic region) is probably useless, and it seems a repetition of what shown in figure 6E.

*Response: The upper part of this panel allows the reader to locate the position of the primers used in the PCR (black or red triangles) and to better understand the PCR amplification bands shown in the lower panel. We have preferred to retain this information.*

Caption of Figure 7. The last sentence about the agarose gel runs can be deleted as it seems unnecessary to me.

*Response: We deleted this sentence.*

P11 L310. blur or blurred?

*Corrected.*

P11 L325-326. I do not think the expectation is that there is no variation in PIP expression agronomic lines. Please consider to avoid this comment.

*Reponse: Actually, this was not so trivial, when discussing with the scientific community, notably when talking about the elite lines. We have preferred to keep this sentence.*

Title head (in bold) of Table 2 is too short (eQTLs)! I suggest to extend it as a full sentence. On the contrary, most of the information provided in the current head should go as table foot note.

*Corrected*

Other portions of Table 2 require attention: for instance 'Nb' is not the correct short version for 'Number', it should be No. (if I am not wrong).

*Corrected*

**Reviewer #2 (Comments for the Author):**

In this article, the authors analyze expression patterns of four plasma membrane-intrinsic proteins (PIPs) across a diversity of European-adapted maize germplasm to identify genomic regions regulating the expression of these genes (eQTLs). Principal component analysis based on heterotic subgroupings revealed differential expression patterns among the selected PIPs, with a distinct separation between B73/Oh43 and PH207-derived germplasm. Subsequent GWAS using several modeling approaches identified a total of 588 eQTLs in proximal and distal locations relative to the genes, with proximal eQTLs contributing the largest, most significant effect. Of the eQTLs identified, a Miniature Inverted-repeat Transposable Element (MITE) insertion in the promoter region of PIP2;5 was present in PH207, but absent in B73 or Oh43, and was selected as a target for additional characterization. Constructs containing the native B73 and PH207 versions of the PIP2;5 promoter driving mVenus, in addition to edited versions of the promoters that inserted or removed the putatively causal MITE, were used to transiently transform maize leaves; confocal microscopy showed a significant reduction in fluorescence intensity whenever the MITE was present, supporting the claim that this mutation underlies the reduced expression of this eQTL. While the findings were exciting, some aspects of the methodology were confusing/questionable and did not produce compelling results.

<u>Major Points:</u>

1. Lines 65-69 are copied almost verbatim from the Hachez et al. (2008) source cited parenthetically in Line 65. Appropriate paraphrasing should be used when referencing source material to avoid plagiarism, however unintentional it may be.

*Response: In fact, this text came from one of our previous publications. We modified these sentences (lines 64-68) to avoid this problem.*

2. The significance levels used for GWAS are inappropriate, with an initial significance threshold arbitrarily set to 5 and then set to 12 for covariate GWAS (Line 564-565; 171-172). If an arbitrary cutoff for significance is chosen, it has generally been considered as p < 5 x 10-8. However, a more suitable approach for establishing significance would be to conduct a Bonferroni correction based on the number of markers used for the association study. In addition, because different significance thresholds are used throughout the manuscript, please make sure to indicate significance thresholds for each test (such as in Figure 8).

*Response: As recommended by the reviewer, the significance threshold was added in the legend of figure 8 and the others. The cutoff of 12 was used to defined the large local eQTLs, not to detect significant SNPs. When highly significant eQTLs were detected, a covariate GWAS was performed for the concerned trait.*

*To detect significant markers, we used a cutoff of 5 so p < 1 x 10-5. Using an arbitrary cutoff of $p < 5 \times 10^{-8}$ would be lower than Bonferroni multiple correction testing threshold ($p < 6.78 \times 10^{-8}$ ). Bonferroni threshold is highly conservative since Bonferronni approach hypothesized that all tests are independent, which are not true since there is linkage disequilibrium (LD) between SNPs. In Gao et al. 2008, Genet Epidemiol. 32(4):361-9 and Gao et al, 2010, Genet Epidemiol. 34(1):100-5 a method was proposed to estimate a number of effective independent test to replace number of tests in Bonferronni approach based on LD matrices. In Negro et al. (2019 BMC Plant Biology, 19:1-22), we estimated this effective number of tests to 191,026 for same genotyping dataset that we used in this study which leads to a threshold of $-log_{10}$(pvalue) = 6,58. However, fixing a lower threshold to $1 \times 10^{-5}$ just means that we tolerated a higher proportion of false positive among the association that we found. Fixing this threshold depends therefore on your objectives since it is a compromise between detecting "true association" (power) while not increasing too much the false association (false positive). Using a threshold of $10^{-5}$ for testing association with 737 202 SNPs means that we expect on average 7.4 false associations among association that we detected for one gene (vs 0.32 using a threshold of $-log_{10}$(pvalue) = 6,58) but using this higher threshold lead to the loss of many putative regulatory regions that could be interesting to better understand architecture of the PIP gene regulation. In this study, we preferred using this threshold to try to catch almost putative regulatory regions of gene expression even if some of this region is false. Strength of association gives, however, an indicator of confidence in this association.*

*We now referred to Negro et al (2019) when mentioning the threshold in Materials & Methods (lines 594-595).*

3. The authors are using RefGen_v2 of the maize genome, which is more than a decade old and several versions outdated. The SNP locations should be migrated to a new version for the publication. No doubt this will clean up analyses, as the authors point out in line 163-166 that LD between distant locations could be caused by assembly error.

*Response: We decided to convert the SNP positions to version 4 to provide a more up-to-date version of our eQTL positions. We changed the MS accordingly. We did not use the v5 because we observed that the gene annotations were more accurate in v4 and this last observation was largely shared by maize communities. For example, we observed that several genes we are interested in are divided in two different genes in v5 or that some genes were mapped at the same location with different gene IDs. This problem is well known in maize community. Since we are working on gene expression, it is important to have a good gene annotation of the genome. We therefore decided to work with the v4 while the v5 is being updated for gene annotation.*

4. How were the 14 lines selected for analysis and inclusion in Table 4? Evaluating the PIP2;5 promoter strength with and without the MITE insertion through use of fluorescence imaging provides evidence (although noisy) to support that this mutation underlies the eQTL. However, Table 4 shows a seemingly clear delineation in PIP2;5 expression levels in the step change between Mo17 and B100. A374 and B97 are below this step change, yet they also have the MITE insertion. Indeed, A374 appears to share the same haplotype of PH207 for all potential polymorphisms. The question is, does the expression across the larger panel correlate with the presence/absence of the MITE?

*Response: The lines were selected for maximizing the Admixture group diversity. The lines included in the Table 4 also include other lines for which the genome was available. This was now mentioned (line 268 and lines 276-277).*

*As mentioned (lines 278-283) and in the discussion (lines 505-511), other indels including the larger ~6 kb indel or other regulatory elements might also contribute to the PIP2;5 local eQTL. This could explain some variations in PIP2;5 expression levels despite the presence of the transposon in lines from the PH207 haplotype, as observed for A374 and B97. Integration of all the polymorphic indels genotyped in a larger panel with the PIP2;5 expression levels will be definitely relevant.*

<u>Minor Points:</u>

1. The authors report that a total of 759,659 markers were selected with a MAF > 0.05 (Line 137, 551-553); however, it is not stated whether all these markers were informative. Are they? Additional information for SNP selection would be useful to understand how they were selected for the analysis.

*Response: In v4, there is a total of 737,202 markers with a MAF > 0.05. This was corrected in the text. These SNPs with MAF>5% were all informative and were all used for performing GWAS for PIP gene expression in two leaf zones.*

2. In the Discussion section the authors need to address the large number of eQTLs identified compared to other studies. Does this relate to false positives or marker selection? This would add to the rationale given in Lines 304-321 is insufficient.

*Response: The large number of local eQTL certainly related more to their linkage disequilibrium with the same causal local polymorphisms since they have a very large effect on gene expression than false positive. Because these local eQTLs explained a very large part of gene expression variation, SNP in low LD with this causal polymorphism could detect them. The removing of these local eQTL by using a multilocus model confirmed that these SNP are not independent (in LD) from the lead SNP. We reformulate the related sentences (Lines 329-341)*

3. Given the ubi-eQTL identified, it seems that the most straight-forward test would be to drop ubi2 as a control gene and rerun the analysis. If the eQTL disappears it is likely an artifacte and the Ubi gene is not a good "control gene". However, if it remains, the authors should reconsider dismissing the eQTL hit on ubiquitin2 in relation to PIP1;1_EZ (Line 188-190. The authors should at least note previous literature linking PIP cycling to the 26S proteasome pathway (see Lee et al., 2009; https://doi.org/10.1105/tpc.108.061994). As PIP1;1 expression is much higher than all other maize aquaporin genes, it is not unreasonable to consider that cycles in protein levels and protein degradation patterns may be differentially regulated than other PIPs, thereby showing a signature at ubi2 even when no other aquaporins do.

*Response: We noticed that PIP1;1 expression of the two subpopulations defined by the allele at the lead SNP was varying as a function of the ubi2 expression (New Figure S3). We calculated the PIP1;1 expression using only Act1 and Ef1α as reference genes and this variation disappeared. We then performed another GWAS as suggested by the reviewer. The ubi-eQTL disappeared suggesting that it was an artifact. However, in order to keep the expression data consistent for the other genes and*

*because PIP1;1 was the only gene concerned by this problem, we decided to include the marker as a covariate in the models for this gene instead of changing the reference genes.*

*The text was modified in light of this new data (lines 197-207).*

4. Line 272-273: Reference is made to an absent Figure 9; should this be Figure 7B?

*Response: Corrected to Figure 8*

5. Line 499: 252 hybrids are reported as included in this panel. Throughout the manuscript reference is made to admixture analysis supporting 6 heterotic groups. List in methods how many lines fell into each of these heterotic groups and whether any groups were over/underrepresented.

*Response: The description of each group was added lines 523-529*

6. Line 512: B104 is an inbred and is otherwise correctly denoted throughout the rest of the manuscript.

*Response: replaced the word “hybrid” by “inbred”*

7. Line 633: Potential typo in reporting microscope model used (Zeiss LSM70).

*Response: corrected*

8. Provide GeneIDs for the different genes discussed (aquaporins, ubi2, etc.), using either B73 RefGen_v3 or Zm-B73-REFERENCE-NAM-5.0 annotations as the highest quality GeneIDs.

*Response: We gave the GeneIDs from v4 as we converted the eQTLs positions in B73 v4.*

02-May-2024
Prof. Dr. Francois Chaumont
UCLouvain
Louvain Institute of Biomolecular Science and Technology
Louvain-la-Neuve B-1348
Belgium

MSID: PP2024-RA-00279D
MS TITLE: Genetic variability of aquaporin expression in maize: from eQTLs to a MITE insertion regulating PIP2;5 expression

Dear Dr. Francois Chaumont:

I am pleased to inform you that your revised manuscript "Genetic variability of aquaporin expression in maize: from eQTLs to a MITE insertion regulating PIP2;5 expression" has been accepted for publication in Plant Physiology. Your manuscript will be checked for consistency with journal formatting and image standards by one of our Science Editors, who will contact you shortly. They may have a number of queries and suggestions for you. Please keep in mind that they work to increase the online visibility of your article and its attraction for readers from the widest of backgrounds. You will have the opportunity to review any revisions, and I hope you will work constructively with them to get the most out of your research. You will also have the opportunity to review all changes when you receive the author proofs for your article.

Once validated for publication, your article will be sent to a Science Editor for a final review of your manuscript and source files. After your final files are uploaded, your article will appear in the next available issue and the online Preview version of your article will be posted after your signed Copyright and Checklist forms and final source files are received.

Please be sure to include the following in your manuscript file:
Harvard style citations
List of all author contributions and funding information at the beginning of manuscript after the titles and author list.
List all supplemental materials associated with the article, with titles, at the end of the ms, before the references and acknowledgments section.
Complete Figure Legends for all figures appearing in the article listed before the Literature Cited.

Thank you and congratulations on your Plant Physiology paper!

Sincerely,
Editor, Plant Physiology

=====
FIRST AUTHOR PROFILES
=====

We recognize the hard work that goes into being First Author of a Plant Physiology paper by publishing a "First Author Feature" for each First Author alongside your paper.

If the first author(s) would like to submit information for a feature on Facebook and Twitter, fill out the form by clicking on the link below:

Plant Physiology First Author Feature Form: https://forms.office.com/r/ZBCGZfhm8F
If you cannot access the form through the link above, please contact Rachel at rbelsky@aspb.org.

To upload an image of yourself to accompany your profile, please put your photo in the correct journal folder at the link below. You must label your image: Author First Name Last Name Article Title.

First Author Profile Image Dropbox: https://www.dropbox.com/scl/fo/xz029p7qjr2jml2rp2kmq/h?dl=0&rlkey=6fkxcaqmxx35ap152b2osy1fz

=====

IMPORTANT REMINDER: PEER REVIEW REPORTS
=====

If you opted to publish a peer review report along with your article during the original submission process, it will be prepared by the editorial staff and publicly posted with your manuscript, inside the zip file that contains any other supplemental material. As a reminder, the peer review report is a public record of all comments from editors and reviewers, as well as your prior responses, as you received them in the decision letters for each draft of your manuscript. If you agreed to publish this report and have changed your mind, or are not sure if you selected this option, please contact the editorial office as soon as possible before signing the license agreement from our publisher.

------------------------------------------------------------------------------ Reviewer comments:
Reviewer #1 (Comments for the Author):

All my comments and requests were satisfied. Indeed, I would like to thank the authors for their accurate and comprehensive answers.

Reviewer #2 (Comments for the Author):

I was Reviewer #2 for the original manuscript. I appreciate the authors incorporating my suggestions in the revised manuscript. It is a very nice manuscript with exciting results.
